# The effects of digital peer support interventions on physical and mental health: a review and meta-analysis

**DOI:** 10.1017/S2045796024000854

**Published:** 2025-02-13

**Authors:** G. Yeo, K. L. Fortuna, J. E. Lansford, K. D. Rudolph

**Affiliations:** 1 N.1 Institute for Health, National University of Singapore, Singapore, Singapore; 2 Dream Big, Ace EL with Dr Yeo, Singapore, Singapore; 3 Geisel School of Medicine at Dartmouth, Hanover, NH, USA; 4 Center for Child and Family Policy, Duke University, Durham, NC, USA; 5 Department of Psychology, College of Liberal Arts and Sciences, University of Illinois Urbana-Champaign, Illinios, USA

**Keywords:** digital health interventions, digital peer support, meta-analysis, physical health, mental health

## Abstract

**Aims:**

Digital peer support interventions have the potential to promote healthy lifestyles and better mental health. This systematic review and meta-analysis synthesizes evidence on the effectiveness of digital peer support interventions for enhancing physical and mental health in healthy individuals rather than those diagnosed with a clinical condition.

**Methods:**

First, we evaluated the impact of digital peer support interventions on physical and mental health outcomes by attending to sources of peer support (informal, naturally occurring peer support; formal support from trained peers), effectiveness demonstrated through different study designs (pre–post comparison vs. well-controlled experimental conditions) and long-term effects of interventions. Second, we examined whether features of digital peer support interventions – specifically, dosage, uptake and platform affordances – moderated intervention effectiveness. Third, we considered moderating effects of individual differences (age and existing health conditions) and country.

**Results:**

Using random-effects modelling, which included 47 studies with 76 effect sizes on physical health, and 73 studies with 118 effect sizes on mental health, we found a moderate effect of digital peer support in improving physical health (standardized mean difference (SMD) = 0.35, *p* < 0.001; 95% CI: 0.30–0.41) and a large effect in enhancing mental health (standardized mean difference(SMD) = 0.53, *p* < 0.001; 95% CI: 0.46–0.61), which were similar across ages and individuals with varying degree of existing health conditions. Different sources of peer support demonstrated similar effects on physical health, but informal, naturally occurring peer support was more effective in bolstering mental health than formal support from trained peers, producing large effects that were comparable to online professional support. Positive effects on physical health were sustained over follow-up assessments, but weakened for mental health over time. Greater dosages of intervention had decreased effectiveness, but uptake of intervention did not moderate the effects on health. Interventions delivered on platforms that afford greater interactivity (apps, social networking sites and video conferencing) were more effective than those with lower interactivity (forums, websites and emails). Digital peer support interventions had stronger effects on improving physical health in Western countries than Eastern countries, but stronger effects on improving mental health in Eastern than Western countries.

**Conclusions:**

Our findings contribute to the nascent conceptual models of digital peer support, lend credence to digital peer support as a scalable preventive intervention with real-world benefits in bolstering individuals’ physical and mental health and provide important insights into best practices.

## Introduction

Rising numbers of deaths are caused by lifestyle factors (Afshin *et al.*, 2017), and poor mental health globally cost $6 trillion in 2023 alone (Health, 2020). Digital technologies are important tools for promoting health and for coping with increasing demands on healthcare systems (WHO, 2019). Human factors, particularly peer support, can increase engagement with and effectiveness of digital interventions in improving unhealthy lifestyle behaviours and mental health (Fortuna *et al.*, 2019; Harding and Chung, 2016). The reciprocal accountability model and qualitative research have attempted to explain why and how peer support provided by digital health interventions is effective (Fortuna *et al.*, 2019; Harding and Chung, 2016). However, there is limited quantitative evidence on the effectiveness of digital peer support interventions for improving physical and mental health (Eysenbach *et al.*, 2004). Existing reviews and meta-analyses of digital peer support interventions involve individuals already diagnosed with clinical conditions (Lloyd-Evans *et al.*, 2014; Patil *et al.*, 2018). Addressing this gap, we evaluated the impact of digital peer support interventions on physical and mental health of healthy support recipients (rather than those who had been diagnosed with a clinical condition).

Our first aim was to assess effects of digital peer support interventions on physical and mental health. Digital peer support interventions involve two possible sources of support (Fortuna *et al.*, 2019): (a) informal, naturally occurring peer support involving individuals who face similar health concerns or share common health-related interests and (b) formal peer support involving trained peer specialists. Although most digital peer support interventions involve one or both of these two sources of peer support (DeBar *et al.*, 2009; Goldberg *et al.*, 2015), few of them distinguish effects of different sources of peer support (Kruzan *et al.*, 2022; Naslund *et al.*, 2014). We also have limited understanding of how sources of peer support compare in their effectiveness with online professional support. Mixed findings also may stem from different methods of assessing effectiveness or from length of follow-up, with some digital peer support interventions demonstrating no long-term effects on physical (Tate *et al.*, 2006) or mental health (Gillard *et al.*, 2022), and others yielding effects lasting many months (Ehlers *et al.*, 2003; Sepah *et al.*, 2017). We hypothesized that digital peer support would enhance physical and mental health, with stronger effects on mental than physical health (Fortuna *et al.*, 2022). We examined how different sources of support differ in their physical and mental health effects, whether stronger inferences can be drawn about the effectiveness of digital peer support interventions from particular study designs, and long-term effects of interventions.

Our second aim was to assess features of digital support interventions that might be related to their effectiveness. Digital peer support interventions have durations from 1 to 144 weeks (Fortuna *et al.*, 2020; Sepah *et al.*, 2017; Watanabe-Ito *et al.*, 2020). Duration (or dosage) of digital health interventions can amplify or weaken effectiveness (Kohl *et al.*, 2013). In addition to dosage, greater uptake of digital peer support interventions (i.e., the percentage of users who completed the intervention) can increase intervention effectiveness (Fortuna *et al.*, 2019), as can affordances of digital platforms (Mohr *et al.*, 2013). New platforms involving mobile apps and social media afford greater interactivity than more conventional platforms, including static websites, videos and emails (Yeo *et al.*, 2024). Digital peer support interventions targeting health are delivered through new (Mamede *et al.*, 2021) and more conventional platforms (Carlsen *et al.*, 2013), warranting attention to how platform affordances with varying interactivity can influence effectiveness. Thus, we examined whether features of digital peer support interventions, specifically, intervention dosage, uptake and platform affordances influence the effects of digital peer support interventions on physical and mental health outcomes.

Our third aim was to examine individual (age and severity of existing health conditions) and country factors that may moderate the effectiveness of digital peer support interventions. Adolescents and young adults spend more time interacting with peers on digital platforms than do children or older adults (Ortiz and Roser, 2019). Adolescents and young adults are also more susceptible to peer influence than are children or older adults (Reiter *et al.*, 2021). Therefore, we hypothesized that digital peer support interventions might be more effective in improving physical and mental health of adolescents and young adults than children and older adults. In addition, the severity of existing health conditions may be an important individual-level moderator. More severe physical and mental health problems may require more intensive intervention than peers are able to provide, including pharmacologic treatments and professional counselling (Qaseem *et al.*, 2024). Thus, we hypothesized that digital peer support interventions may be more effective for individuals who are relatively healthy rather than those experiencing more severe health problems. At the country level, we compared Eastern (generally more collectivist) and Western (generally more individualist) countries as this distinction has been a cornerstone of theoretical work on cultural values in psychological science (Hofstede Insights, 2023; Kâğıtçıbaşı, 1997; Triandis *et al.*, 1986). For example, relative to their Western counterparts, Eastern (largely Asian) individuals value emotional self-control and dealing with mental health problems on their own rather than through self-disclosure (Lee *et al.*, 2014). Mental health problems are also stigmatized more in some countries than others (Misra *et al.*, 2021). Thus, we examined whether country (Western vs. Eastern) moderates the effectiveness of digital peer support interventions.

## Method

This meta-analysis was conducted according to the guidelines from Cooper et al. (2019) and reported based on the latest version of Preferred Reporting Items for Systematic Reviews and Meta-Analyses guidelines (PRISMA 2020; Page *et al.*, 2021). A protocol was registered *a priori* following the PRISMA guideline (PROSPERO registration number CRD [Blinded]). All data, analysis code and research materials are available at [Blinded]. Data were analyzed using R studio version 4.0.0, with the ‘metafor’ and ‘robumta’ packages version 3.02 (Fisher *et al.*, 2017; R Development Core Team, 2017; Viechtbauer, 2010).

With the assistance of a staff librarian at the first author’s affiliated institution, two research assistants independently used two search strategies to systematically collect academic articles reporting on digital peer and professional support interventions conceptualized and operationalized as active engagement of (a) informal, naturally occurring peer support, (b) formal unpaid and/or paid peer support and/or (c) professional support. First, a systematic search was conducted in December 2023 across PsycINFO, EMBASE and CINAHL because these data bases cover two fields pertinent to this review – social science and medical research. Searches were rerun just before the final analyses in May 2024 to include further articles published in the intervening months. Searches were limited to academic articles, human studies and papers written in English or with an English translation. Second, reference lists of the included studies (including other reviews and meta-analyses) were searched manually and cross-referenced for additional articles. Table A1 and A2 (Online Supplement) detail the search terms developed using the population, intervention, comparison, outcome search strategy and the full search strings.

Study screening was conducted using Covidence software (Veritas Health Innovation, Melbourne, Australia). Figure A7 (Online Supplement) presents a flowchart of the study selection and screening processes. Inclusion and exclusion criteria were established *a priori* (Online Supplement Table A1). Two research assistants extracted and coded data on sample characteristics, physical and mental health outcomes, active engagement of digital support from different sources, and means, standard deviations and odd ratios to calculate SMD/Cohen’s D (Online Supplement Tables A3–A6). Coders achieved 83% agreement on their codes. Any discrepancies in coding were resolved by a discussion. Studies were independently evaluated for quality by two reviewers, with differences discussed and resolved using the Cochrane Collaboration’s risk of bias tool (Higgins *et al.*, 2022); results are reported in Online Supplement Tables A5–A6. If studies contributed multiple dependent effect sizes because they examined more than one source of support, included more than one indicator of physical or mental health, or included both Western and Eastern countries, we dealt with this in three ways that are explained in Online Supplement Tables A5–A6. Pooled effect sizes of SMD were used as the effect sizes in assessing intervention effects on physical and mental health. We conducted subgroup analyses to assess effectiveness of different sources of peer support and to assess effectiveness based on whether outcomes were measured immediately post-intervention versus long-term. We also tested for risk of publication bias.

## Results

Summary and sample statistics of included digital peer support intervention studies are presented in Online Supplement Tables A3–A6. Forty-seven of the studies examined physical health and produced 76 effects sizes, and 73 of the studies examined mental health and produced 118 effect sizes (Figure A7 in Online Supplement). Given heterogeneity observed among the results of the studies, we used a random-effects model in conducting meta-analyses and meta-regressions (Higgins *et al.*, 2003; Table 1). For physical and mental health separately, we included a forest plot to visualize the effect sizes and confidence intervals from the included studies, with a computed summary effect size (Figure A8 in Online Supplement). Small effect sizes are below 0.30, moderate effect sizes are between 0.30 and 0.50, and large effect sizes are above 0.50 (Cohen, 1992).

**Table 1.** Digital peer support interventions and physical and mental health

	Digital peer support intervention					
Outcomes	*k*	*N*	*r*	95% CI	*Q*	*I*^2^
i. Physical health	76	145326	0.35**	(0.30, 0.41)	32258.22	99
			(0.19–0.88)			
ii. Mental health	118	52703	0.53**	(0.46, 0.61)	7182.52	98.87
			(0.01–0.98)			

*Note: k* = number of effect sizes; *N* = sample size; *r* = effect sizes (range of mean effect sizes); CI = confidence interval; *Q=*the ratio of observed variance to within-study variance; *I*^2^ = percentage of observed variation that can be attributed to the actual differences between studies, rather than within-study variance.

The first aim was to test effects of digital peer support interventions on physical and mental health. Digital peer support interventions had moderate effects in improving physical health, SMD = 0.35, *p* < 0.001 (95% CI: 0.30–0.41) (row i Table 1), and large effects in enhancing mental health, SMD = 0.53, *p* < 0.001 (95% CI: 0.46–0.61) (row ii Table 1), with larger effects on mental than physical health, *Q*_between_ = 5.90, *p* = 0.015 (row Ai Table 2).

**Table 2.** Moderators of digital peer support interventions and physical and mental health

	Physical and mental health				
Moderator	*k*	SMD	95% CI	Between group *Q*	*I*^2^
A. Outcome					
i. Physical health		0.35 (0.19–0.88)	(0.30, 0.41)	5.92*	99.22
ii. Mental health		0.53 (0.01–0.98)	(0.46, 0.61)		
B. Sources of digital peer support					
i. Physical health					
Informal naturally occurring	76	0.34 (0.03–0.83)	(0.25, 0.43)	3.17	95.43
Formal unpaid and/or paid		0.38 (0.02–0.55)	(0.13, 0.64)		
Professional support		0.27 (0.05–0.50)	(0.06, 0.61)		
Combination of peer and professional support		0.46 (0.06–0.89)	(0.21, 0.74)		
ii. Mental health					
Informal naturally occurring	118	0.74 (0.01–0.98)	(0.52, 0.85)	13.90**	98.74
Formal unpaid and/or paid		0.37 (0.01–0.79)	(0.15, 0.60)		
Professional support		0.60 (0.03–0.95)	(0.10, 0.78)		
Combination of peer and professional support		0.57 (0.01–0.97)	(0.12, 0.70)		
C. Study design					
i. Physical health					
Pre vs. post intervention	74	0.42 (0.05–0.89)	(0.34, 0.51)	5.39	98.09
Treatment (digital peer support) vs. control		0.29 (0.02–0.83)	(0.05, 0.51)		
Treatment vs. alternative digital interventions (e.g., online psychoeducation)		0.21 (0.02–0.60)	(0.11, 0.52)		
ii. Mental health					
Pre vs. post intervention	118	0.67 (0.01–0.98)	(0.49, 0.85)	2.59	98.74
Treatment (digital peer support) vs. control		0.58 (0.04–0.98)	(0.12, 0.84)		
Treatment vs. alternative digital interventions (e.g., online psychoeducation)		0.41 (0.01–0.93)	(0.10, 0.80)		
	Physical and mental health				
Moderator	*k*	SMD	95% CI	Between group *Q*	*I*^2^
D. Post-intervention vs. follow-up					
i. Physical health					
Post intervention assessment	75	0.37 (0.02–0.89)	(0.30, 0.44)	0.07	99.00
Follow-up assessment		0.35 (0.03–0.56)	(0.11, 0.59)		
ii. Mental health					
Post intervention assessment	118	0.54 (0.01)	(0.43, 0.64)	13.08**	99.18
Follow-up assessment		0.37 (0.98)	(0.46, 0.50)		
E. Length of follow-up assessment					
i. Physical health	15	0.0002 (0.02–0.83)	(−0.0057, 0.0062)	0.01	98.68
ii. Mental health	49	−0.10 (0.01–0.98)	(−0.22, −0.001)	8.56*	98.48
F. Dosage					
i. Physical health	75	−0.0027 (0.02–0.89)	(−0.0051, −0.0002)	4.60*	98.85
ii. Mental health	118	−0.0068 (0.01–0.98)	(−0.013, −0.0007)	4.70*	99.11
G. Uptake					
i. Physical health	76	−0.0002 (0.02–0.89)	(−0.0037, 0.0033)	0.01	98.65
ii. Mental health	95	0.0017 (0.01–0.98)	(−0.0036, 0.0070)	0.41	99.13
H. Platform type					
i. Physical health					
Apps	76	0.38 (0.04–0.70)	(0.10, 0.47)	11.53*	96.97
Forums, discussion boards		0.20 (0.03–0.83)	(0.10, 0.26)		
Social networking sites		0.35 (0.05–0.83)	(0.14, 0.50)		
Websites		0.26 (0.02–0.77)	(0.15, 0.37)		
Video conference		0.34 (0.2–0.89)	(0.12, 0.68)		
Others (e.g., emails, text messages, and phone calls)		0.24 (0.02–0.56)	(0.11, 0.30)		
	Physical and mental health				
Moderator	*k*	SMD	95% CI	Between group *Q*	*I*^2^
ii. Mental health					
Apps	118	0.42 (0.06–0.96)	(0.19, 0.65)	18.96**	99.05
Forums, discussion boards		0.21 (0.01–074)	(0.12, 0.55)		
Social networking sites		0.34 (0.01–0.93)	(0.19, 0.69)		
Websites		0.13 (0.01–0.98)	(0.05, 0.41)		
Video conference		0.41 (0.16–0.95)	(0.004, 0.82)		
Others (e.g., emails, text messages, and phone calls)		0.12 (0.01–0.74)	(0.04, 0.21)		
I. Age					
i. Physical health	69	−0.0001 (0.02–0.89)	(−0.0043, 0.0042)	0.001	98.32
ii. Mental health	101	0.006 (0.01–0.98)	(−0.0013, 0.0133)	2.56	99.17
J. Severity of existing conditions					
i. Physical health	16	0.0003 (0.02–0.63)	(−0.008, 0.009)	0.01	97.24
ii. Mental health	88	−0.0020 (0.01–0.98)	(−0.013, 0.009)	0.13	99.15
K. Cultural contexts^a^					
i. Physical health					
Western	76	0.52 (0.02–0.89)	(0.27, 0.72)	7.53*	98.61
Eastern		0.32 (0.20–0.54)	(0.26, 0.49)		
ii. Mental health					
Western	118	0.27 (0.01–0.98)	(0.12, 0.35)	12.29**	99.04
Eastern		0.52 (0.06–0.96)	(0.41, 0.63)		
A mix of cultural contexts		0.39	(0.18, 0.55)		

a There were two effect sizes for physical health and six effect sizes for mental health from studies that examined a mix of Western and Eastern cultural contexts, and we did not include them. Following the guideline outlined by Borenstein et al. (2021), meta-regression involving subgroup analyses requires at least *k* > 10.

*Note: k* = number of effect sizes; *N* = sample size; *r* = effect sizes (range of mean effect sizes); CI = confidence interval; In conducting meta-regressions, the *Q* test is used to determine if the subgroup differences are significant – whether the observed values of *Q* (i.e., between group *Q*) are greater than sampling error by comparing the observed values of *Q* to the critical/expected values from a *χ*^2^ distribution (Borenstein et al., 2021; Harrer et al., 2021). When the observed values of *Q* are larger than the expected ones (based on the *χ*^2^ distribution), the *p*-value will be significant, and this indicates that the effect sizes between the subgroups are different. The *Q* test is an omnibus test, which tests the null hypothesis that effect sizes across subgroups are comparable, and is significant when at least two subgroups or any combinations thereof, are different (Borenstein et al., 2021; Harrer et al., 2021).

*I*^2^ = percentage of observed variation that can be attributed to the actual differences between studies, rather than within-study variance.

* *p* < 0.05; **p < 0.001.

We performed subgroup analyses using meta-regressions to compare the effects based on the source of digital support^1^ (informal, naturally occurring peer support, formal unpaid and/or paid peer support, professional support and a combination of both professional and peer support). Different sources of support had comparable moderate effectiveness in bolstering physical health, *Q*_between_ = 3.17, *p* = 0.367 (row Bi Table 2). In contrast, different sources of support displayed significant differences in their effects on mental health, *Q*_between_ = 13.90, *p* = 0.001 (row Bii Table 2); specifically, informal, naturally occurring peer support, SMD = 0.74, *p* < 0.001 (95% CI: 0.52–0.85) demonstrated comparable effectiveness as professional support, SMD = 0.60, *p* < 0.001 (95% CI: 0.10–0.78), and a combination of professional and peer support, SMD = 0.57, *p* < 0.001 (95% CI: 0.12–0.70), in bolstering mental health with large effect sizes, which were greater than the moderate positive effects of formal unpaid and/or paid peer support, SMD = 0.37, *p* < 0.001 (95% CI: 0.15–0.60).

We next compared the effects of digital peer support interventions on physical and mental health across different study designs (i.e., pre- vs. post-intervention, wait-list control and comparisons with alternative digital interventions). Different study designs showed comparable effects on physical health, *Q*_between_ = 5.39, *p* = 0.068 (row Ci Table 2), and mental health, *Q*_between_ = 2.59, *p* = 0.274 (row Cii Table 2). Subgroup comparisons of digital peer support interventions that assessed mental and physical health immediately post-intervention and those with longer-term follow-up assessments indicated comparable effects on physical health, *Q*_between_ = 0.07, *p* = 0.789 (row Di Table 2), but their effects were stronger for immediately post-intervention than longer-term follow-up assessments for mental health, *Q*_between_ = 13.08, *p* = 0.001 (row Dii Table 2). Longer durations of follow-ups attenuated the positive effects of digital peer support interventions on mental health, *Q*_between_ = 8.56, *p* = 0.0034 (row Eii Table 2), but not on physical health, *Q*_between_ = 0.01, *p* = 0.934 (row Ei Table 2).

The second aim was to assess whether features of digital support interventions moderated their effects. Duration of digital peer support interventions influenced effectiveness for physical health, *Q*_between_ = 4.60, *p* = 0.032 (row Fi Table 2) and mental health, *Q*_between_ = 4.70, *p* = 0.030 (row Fii Table 2); interventions of longer duration had decreased effectiveness on physical health, SMD = −0.0027, *p* < 0.001 (95% CI: −0.0051 to −0.0002), and mental health SMD = −0.0068, *p* < 0.001 (95% CI: −0.013 to −0.0007). Uptake of digital peer support interventions (the percentage of users who completed the intervention ranged from 18.5–100% for physical health and 13.6–100% for mental health) did not moderate the effect of the interventions on physical health, *Q*_between_ = 0.01; *p* = .906 (row Gi Table 2), or mental health, *Q*_between_ = 0.41; *p* = .524 (row Gii Table 2).

Regarding platform affordances, *Q*_between_ = 11.53; *p* = .0418 (row Hi Table 2), the moderate effect of digital peer support interventions on physical health for apps; SMD = 0.38, *p* < 0.001 (95% CI: 0.10–0.47), social networking sites; SMD = 0.35, *p* < 0.001 (95% CI: 0.14–0.50) and video conference; SMD = 0.34, *p* < 0.001 (95% CI: 0.12–0.68), was greater than the small effect for forums and discussion boards; SMD = 0.20, *p* < 0.001 (95% CI: 0.10–0.26), websites; SMD = 0.26, *p* < 0.001 (95% CI: 0.15–0.37) and others (e.g., emails, text messages and phone calls); SMD = 0.24, *p* < 0.001 (95% CI: 0.11–0.30). Similarly, the moderate effect size of digital peer support interventions on mental health, *Q*_between_ = 18.96; *p* < .001 (row Hii Table 2) for apps; SMD = 0.42, *p* < 0.001 (95% CI: 0.19–0.65), social networking sites; SMD = 0.34, *p* < 0.001 (95% CI: 0.19–0.69) and video conference; SMD = 0.41, *p* < 0.001 (95% CI: 0.004–0.82), was greater than the small effects for forums and discussion boards; SMD = 0.21, *p* < 0.001 (95% CI: 0.12–0.55), websites; SMD = 0.13, *p* < 0.001 (95% CI: 0.05–0.41), and others (e.g., emails, text messages and phone calls); SMD = 0.12, *p* < 0.001 (95% CI: 0.04–0.21).

The third aim was to assess individual and country moderators. Contrary to our hypotheses, age did not moderate the impact of digital peer support interventions on physical health, *Q*_between_ = 0.001, *p* = 0.980 (row Ii Table 2) or mental health, *Q*_between_ = 2.56, *p* = 0.110 (row Iii Table 2). Also contrary to our hypotheses, the effect of digital peer support interventions on physical health, *Q*_between_ = 0.01, *p* = 0.938 (row Ji Table 2), and mental health, *Q*_between_ = 0.13, *p* = 0.714 (row Jii Table 2), did not attenuate with increasing severity of existing physical and mental health conditions.

Studies included in the meta-analyses involved 14 countries, but there were not enough studies from each country to allow for a comparison of effect sizes among individual countries. Instead, we followed the common approach of comparing Western (e.g., Australia, Canada, Germany, United States) and Eastern (e.g., China, Pakistan, Singapore, Taiwan) countries (Yeo *et al.*, 2024). Digital peer support interventions conducted in Western and Eastern countries differed significantly in effects on physical health, *Q*_between_ = 7.53, *p* = 0.02 (row Ki Table 2) and mental health, *Q*_between_ = 12.29, *p* = 0.0005 (row Kii Table 2). Digital peer support interventions had greater effects on improving physical health in Western countries, SMD = 0.52, *p* < 0.001 (95% CI: 0.27–0.72) than Eastern countries, SMD = 0.32, *p* < 0.001 (95% CI: 0.26–0.49). In contrast, digital peer support interventions had greater effects on increasing mental health in Eastern countries, SMD = 0.52, *p* < 0.001 (95% CI: 0.41–0.63) than Western countries, SMD = 0.27, *p* < 0.001 (95% CI: 0.12–0.35).

For physical and mental health separately, three analyses were used to ascertain publication bias and are presented in Figure A9 (Online Supplement). We conclude from these analyses that there is an absence of publication bias for studies included in our meta-analyses.

## Discussion

This systematic review and meta-analysis synthesized findings on the implications of digital peer support interventions for physical and mental health. In relation to our first aim, we found that digital peer support interventions had particularly strong positive effects on mental health that were larger than the moderate positive effects on physical health. We also found that informal, naturally occurring peer support that involves peers who share similar physical and mental health concerns was the most promising in improving mental health, with large effect sizes that were comparable to the effects of professionals, such as therapists, psychologists and counsellors, providing online support. In contrast, digital interventions with different sources of peer support conferred similar moderate benefits to physical health. Support involving individuals with common lived experiences or interests in mental health that is less structured and delivered in informal, naturally occurring contexts reduces stigma, facilitates self-disclosure, builds therapeutic alliances that empower individuals, and increases self-esteem and self-efficacy (Fortuna *et al.*, 2019, 2020). These conditions may contribute to mechanisms of action (e.g., more effective coping, support seeking efforts) that enhance mental health, in particular (Wolpert *et al.*, 2021). Because less stigma is associated with physical health problems, different sources of digitally delivered peer support may function in the same manner with similar mechanisms of action and impacts. These results provide important insights into the best practices in digital peer support and are consistent with the movement towards informal, naturally occurring peer-to-peer virtual groups (Eysenbach *et al.*, 2004).

Our findings contrasted with those from one systematic review on digital peer support interventions for individuals with serious mental illnesses (Fortuna *et al.*, 2020), which found that formal peer support involving peer specialists who are trained and/or participate in consumer or peer-run programmes are most effective. It is possible that individuals with serious mental health problems require more intensive intervention involving peer specialists than peers with common lived experiences are able to provide (Qaseem *et al.*, 2024). Thus, digital interventions with informal, naturally occurring peer support may be more effective for individuals who are relatively healthy rather than those experiencing significant health problems.

When comparing different methodological approaches, we found that in addition to improving health after as compared to before the intervention, digital peer support effectively improves health as compared to control conditions and other digital interventions, such as online psychoeducation. A common concern about digital health interventions is whether they have long-lasting effectiveness (McCool *et al.*, 2020), especially for digitally delivered peer support where the effectiveness may be short-lived (Gillard *et al.*, 2022; Tate *et al.*, 2006). Our results revealed that digital peer support interventions conferred short- and long-term physical health benefits, such as reducing obesity and high blood pressure as well as improving sleep and dietary habits (Sepah *et al.*, 2017). In contrast, the effects of digital peer support intervention for mental health became weaker with longer follow-up assessments. The long-term effects of digital peer support interventions on physical health, which averaged 34.1 weeks in this synthesis, suggest that digital peer support interventions may have adaptive functions that influence other domains of function (e.g., lifestyle behaviours) that extend over time to promote positive health. For mental health, because earlier findings revealed that informal, naturally occurring digital peer support is most effective, perhaps only this form of digital peer support has more sustained effects on mental health – a notion that warrants future research attention.

In relation to our second aim involving features of digital peer support interventions, unexpectedly, our synthesis found that greater dosage of digital peer support interventions decreases their effectiveness in bolstering both physical and mental health. Perhaps extended periods of intervention result in negative interactions with peers involving co-rumination, cyberbullying and trolling that can negatively impact physical and mental health (Naslund *et al.*, 2014). Randomized control trials that examine the effects of varying dosages are warranted to elucidate the optimal dose for improving physical and mental health. Of note, uptake of the interventions, reflected in the percentage of users who completed them, did not moderate their impact on physical and mental health. Studies of facilitators of and barriers to the uptake of digital behavioural health interventions argue that engagement affects intervention effectiveness (Borghouts *et al.*, 2021). Our review and meta-analysis found limited work on the implementation effectiveness of digital peer support interventions involving engagement and uptake (Sepah *et al.*, 2017; Suffoletto *et al.*, 2021), and this small body of work was focused primarily on attrition, indicating a need for future studies to consider other engagement indicators that demonstrate participation in interventions. For instance, studies could consider the extent of peer support accessed (e.g., number and duration of peer interactions) or engagement in intervention-related activities (e.g., specific activities with peers; Kruzan *et al.*, 2022).

We also found that the effect of digital peer interventions on physical and mental health was greater on new interactive platforms, such as mobile apps, social networking sites and video conferencing, than more conventional, less interactive platforms, including websites, forums and emails. Although there are numerous studies examining affordances of digital platforms, such as asynchronicity (Mohr *et al.*, 2013), we have a limited understanding of how specific platform affordances interact with peer support to impact physical and mental health. Future work on digital peer support interventions should consider different platform affordances and their interactions with peer support in modulating effectiveness.

Our third aim involved examining whether individual (age and severity of existing health conditions) or country differences moderated the effectiveness of these interventions. Although we hypothesized that these interventions may be more effective in adolescents and young adults than in children or older adults, given the susceptibility of the former group to peer influence (Reiter *et al.*, 2021) and their comfort with, and frequency of use of, digital platforms (Ortiz and Roser, 2019), age did not significantly moderate the effects. Moreover, findings did not support our hypothesis that digital peer support interventions would be less effective for individuals with more severe existing health conditions, who may require more intensive intervention than peers are able to provide (Qaseem *et al.*, 2024), than relatively healthy individuals. Thus, results of the meta-analysis support the use of digital peer support interventions across different developmental stages and populations, although certain types of digital peer interventions may be less effective for individuals with more severe, diagnosable health problems, as discussed earlier.

Eastern versus Western countries did differ in the effectiveness of digital peer support intervention, such that individuals from Eastern countries benefited more in terms of mental health, whereas individuals from Western countries benefited more in terms of physical health. Because Eastern countries may be less open to addressing mental health problems (Lee *et al.*, 2014), individuals from these countries may be reluctant to disclose mental health struggles to significant others and therefore lack opportunities to receive support from family, friends or romantic partners; these individuals may therefore benefit from opportunities to receive online peer support. However, because discussing physical health concerns is more acceptable in these countries, individuals may be less in need of digital peer support for optimizing physical health. Further understanding the mechanisms underlying these country differences will be essential for further refinement of digital peer support interventions to enhance their generalizability across countries.

There are three important limitations of our systematic review and meta-analysis. First, we found that digital interventions combine peer support with different treatment components, such as online psychoeducation (Suffoletto *et al.*, 2021). Rigorous intervention designs using multiphase optimization strategy, for instance sequential multiple-assignment randomized trial, is needed to distinguish the impact of peer support from other active treatment components (Murray *et al.*, 2016), so that peer support can be supplemented as necessary. Second, the observed heterogeneity in findings across studies suggests that there may be important moderators of effectiveness that need to be considered, such as the settings (e.g., hospitals, research institutes, community) in which the digital peer support interventions were implemented.

Finally, digital peer support interventions were mostly found in Western countries (38/47, 80.9% for physical health and 63/73, 86.3% for mental health), and only half of the studies (22/47, 46.8% for physical health and 43/73, 58.9% for mental health) reported information on the racial or ethnic composition of their samples. When information was available, the samples were predominantly White (ranged from 49.1%% to 88%) with limited racial or ethnic diversity. As such, our findings may not be generalizable to other geographic regions or demographic groups. In light of racial and ethnic marginalization and health disparities, future digital peer support interventions for health that address racial or ethnic disparities, particularly the evaluation of culturally appropriate interventions, are needed (Ellis *et al.*, 2022).

In conclusion, this review and meta-analysis advances understanding of effective digital peer support for healthy populations beyond previous reviews and meta-analyses that focused on individuals already diagnosed with clinical conditions (Lloyd-Evans *et al.*, 2014; Patil *et al.*, 2018). Our findings provide important insights into best practices for evidence-based digital peer support interventions. Digital peer support interventions are related to improved mental and physical health outcomes across ages, for individuals differing in initial severity of health conditions and across countries.

## Supporting information

Yeo et al. supplementary material 1 Yeo et al. supplementary material

Yeo et al. supplementary material 2 Yeo et al. supplementary material

Yeo et al. supplementary material 3 Yeo et al. supplementary material

## Data Availability

Openscience framework (OFS Home): https://osf.io/jzwsg/.

## Footnotes

1 Although the technology-based models of peer support suggests formal peer support with peers who are trained and support from consumer/peer-run programmes are distinct, most digital peer support interventions do not make reference to this model or make such a distinction. Interventions often involve peer supporters who are trained and they may or may not be from consumer/peer-run programmes. Thus, in this review and meta-analysis, we consider them collectively as formal peer support.

## Supplementary material

The supplementary material for this article can be found at https://doi.org/10.1017/S2045796024000854.
