# Supplementary material for: The effects of digital peer support interventions on physical and mental health: a review and meta-analysis
Source: Epidemiol Psychiatr Sci. 2025 Feb 13;34:e9. doi: 10.1017/S2045796024000854 (PMC11886969; doi:10.1017/S2045796024000854)
Supplement: Yeo et al. supplementary material 1 — Yeo et al. supplementary material [file S2045796024000854sup001.docx]

Figure A7

*PRISMA Flow Diagram of Search Strategy on Identification and Screening of Studies, and the Finalised Number of Articles for Meta-Analysis*

**Identification of studies via databases and registers**

Duplicate records removed

(n = 33,056)

Records identified from databases (n =94,443)

**Identification**

Records screened

(n = 61,387)

Records excluded

(n = 60,387)

Records sought for retrieval (n = 1,387)

Records not retrieved

(n = 98)

**Screening**

Full-text articles excluded:

Wrong study design (n = 340)

Insufficient statistics to compute effect size (n = 98)

Wrong population (n = 198)

Wrong interventions (n = 501)

Did not have English translation (n=32)

Full-text articles assessed for eligibility (n = 1,289)

**Included**

Studies included in review

(n = 120)

Note. Of the 94,443 studies identified in the initial searches, 1,289 full-text articles selected for full text review were independently screened by another two reviewers (undergraduate research interns) with any discrepancies resolved through consensus. A third reviewer was contacted if consensus could not be reached.

Figure A8

*Forest Plot of Data Investigating Digital Peer Support Interventions with Physical and Mental Health*

Physical Health

Author(s), Year Standardized Mean Difference [95% CI]
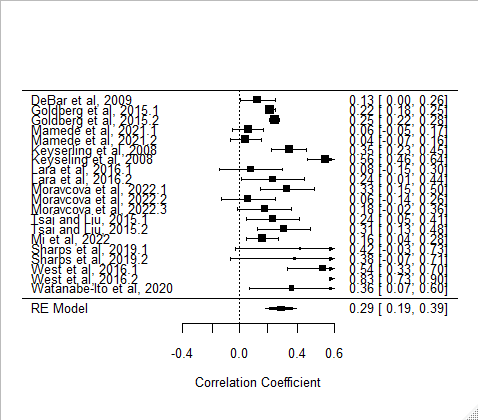


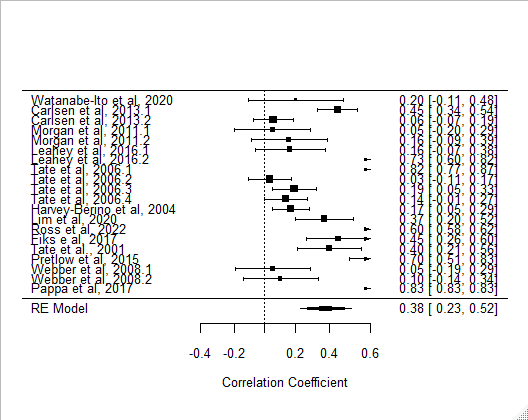


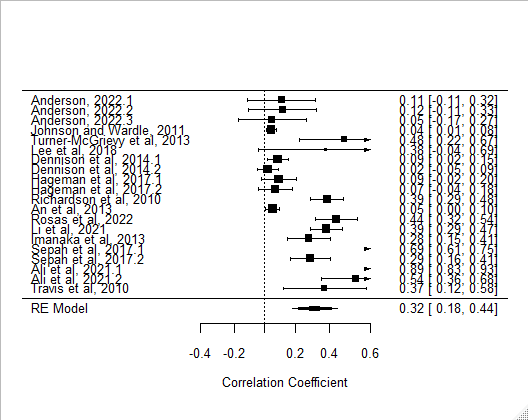


[Continue from above]


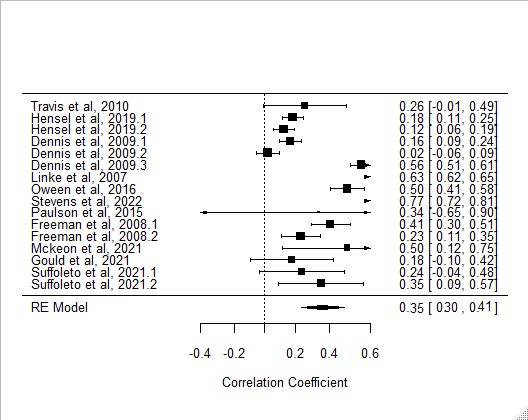
Author(s), Year Standardized Mean Difference [95% CI]

Standardized Mean Difference

Mental Health

Author(s), Year Standardized Mean Difference [95% CI]


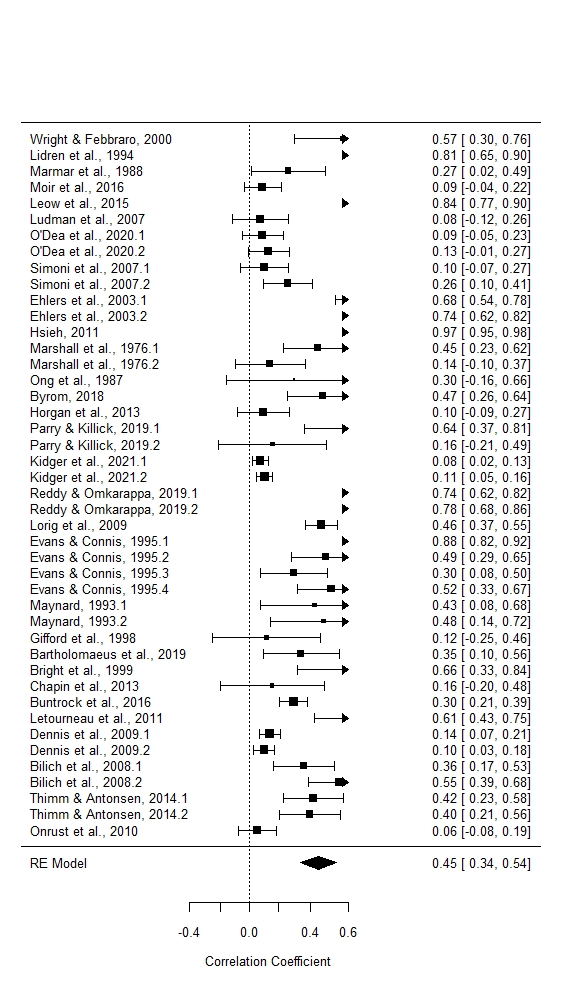


[Continue from above]

Author(s), Year Standardized Mean Difference [95% CI]
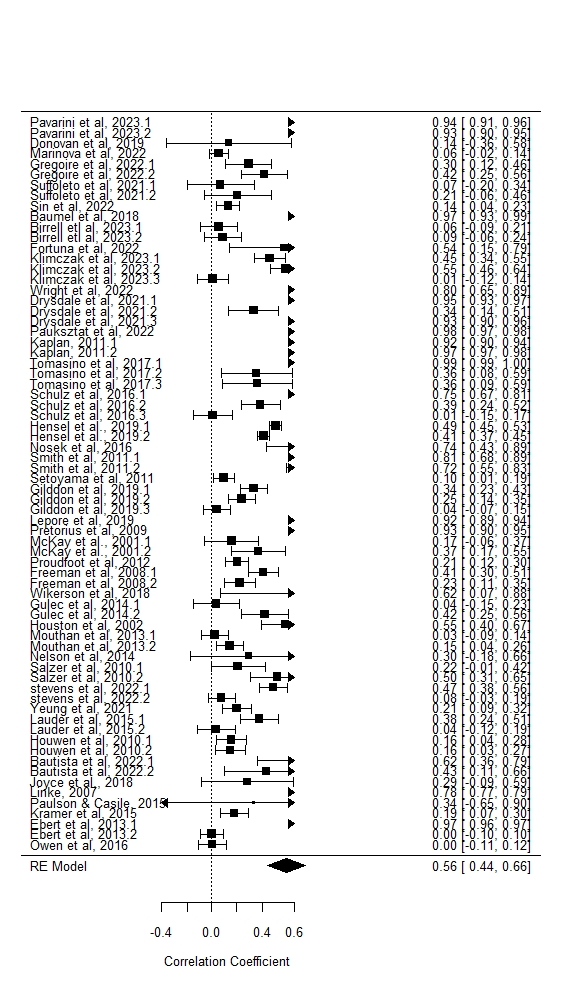


[Continue from above]

Author(s), Year Standardized Mean Difference [95% CI]


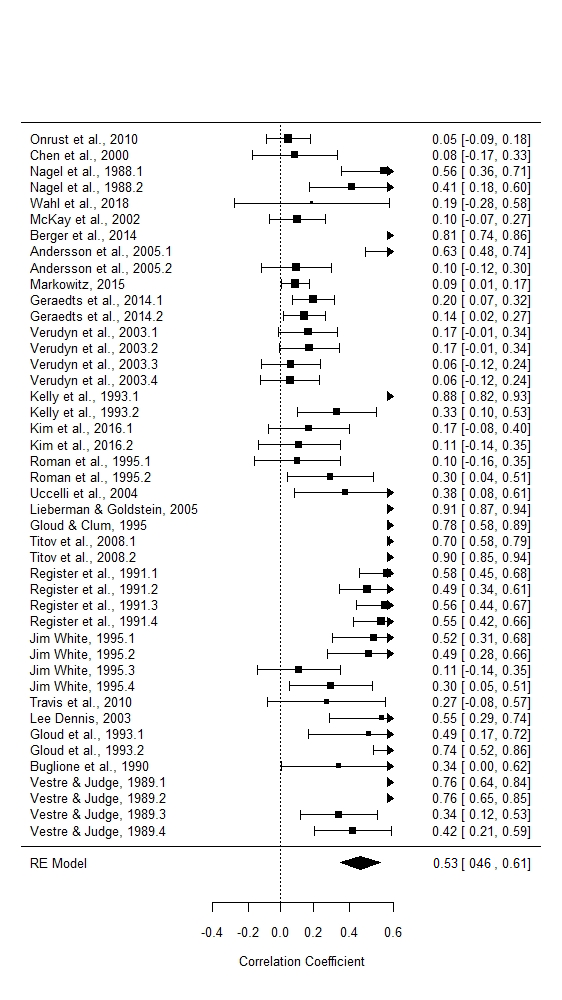


Standardized Mean Difference

*Note.* In the plot, each study is represented by a point estimate and is bounded by a CI for the effect, and at the bottom of the plot, the summary effect size is represented by the polygon, with its width representing 95% CI. Studies with larger squares contributed more to the summary effect size as compared to other studies. Small effect sizes refer to those below 0.30, moderate effect sizes are between 0.30 and 0.50, and large effect sizes are above 0.50 (Cohen, 1992).

Figure A9

*Funnel Plot of the Effect Sizes of Digital Peer Support Interventions with Physical and Mental Health*

Physical Health


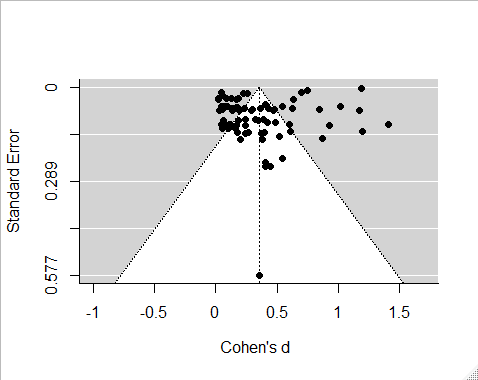


Mental Health


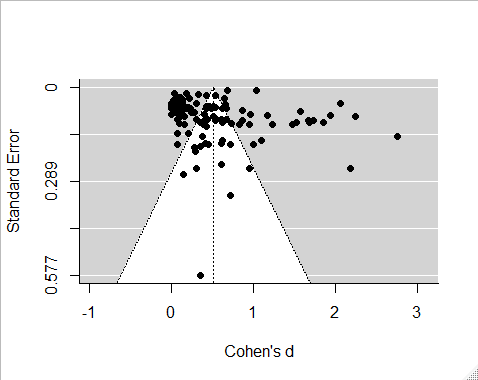


Note. The funnel lines indicate the degree of spread that is expected for a given level of standard error and are centered on the summary effect size that is represented by the vertical line. Data points scattered symmetrically on both sides of the funnel line and having the shape of an even funnel provide evidence for an unbiased sample. The funnel plot analyses reveal possible publication bias that is evident by a cluster of data that is non-symmetrical and deviates from the shape of a funnel. The points that fell into the grey area were asymmetrical and indicated that publication bias may exist. Because funnel plots offer only a subjective measure of potential publication bias (Egger et al., 1997), we also used the rank correlation and the Egger’s regression tests, which are objective measures of potential bias (Egger et al., 1997). These tests revealed non-significant results, which confirmed an absence of publication bias for studies included in our meta-analyses.
