# Supplementary material for: The effects of digital peer support interventions on physical and mental health: a review and meta-analysis
Source: Epidemiol Psychiatr Sci. 2025 Feb 13;34:e9. doi: 10.1017/S2045796024000854 (PMC11886969; doi:10.1017/S2045796024000854)
Supplement: Yeo et al. supplementary material 2 — Yeo et al. supplementary material [file S2045796024000854sup002.docx]

**Online Supplement**

Table A1

Search Terms Used for the Formulation of the Search Strings in the Systematic Search

| **PICO concept** | **Search Terms** |
| --- | --- |
| **Population** | Healthy individuals receiving digital peer support, whether informally or formally |
| **Intervention (or Exposure)** | Digital peer support |
| **Comparison** | Digital professional support (therapists, clinicians, and psychologists) |
| **Outcome** | Mental health (e.g., anxiety, depression, mental well-being, psychological well-being, life satisfaction, distress, loneliness, internalizing symptom, and externalizing symptom)  Physical health (e.g., weight management, obesity, life style behaviour and/or promotion, physical activity, sleep, dietary intake, eating habits, BMI, blood pressure, and glucose level) |
| **Inclusion and exclusion criteria were established *a priori*** | |
| **Inclusion** | (a) be a full-text empirical study written in English;  (b) healthy individuals receiving digital peer support, whether informally or formally  (c) provide sufficient statistics for calculation of effect sizes; interventions needed to list sample sizes, means, standard deviations (SDs), and/or odds-ratios. For studies that lacked statistical information needed to calculate effect sizes, authors were contacted to provide the missing data;  (d) interventions needed to have at least one active engagement of digital support: informal naturally occurring peer support (peers who face similar mental and physical health concerns and/or common health related interests; Fortuna et al., 2019), formal unpaid and/or paid peer support (peers who are trained and/or participate in consumer or peer-run program e.g., community volunteers/health workers and coaches; Fortuna et al., 2019), and professional support (therapists, clinicians, and psychologists);  (e) include at least one measure of mental health (e.g., anxiety, depression, mental well-being, psychological well-being, life satisfaction, distress, loneliness, internalizing symptom, and externalizing symptom) and/or physical health (e.g., weight management, obesity, life style behaviour and/or promotion, physical activity, sleep, dietary intake, eating habits, BMI, blood pressure, and glucose level) |
| **Exclusion** | a) wrong study designs (e.g., qualitative studies, theory or protocol papers for intervention) with no relevant statistics for the calculation of effect sizes (340 articles);  (b) did not report sufficient statistics required to compute effect sizes (authors of these studies failed to respond to requests for the needed information; 98 articles);  (c) did not have full-text available (14 articles);  (d) wrong population (e.g., individuals with clinical conditions; 198 articles);  (e) wrong interventions (e.g., digital peer supporters’ training; 501 articles);  (f) did not have English translations (32 articles) |

*Note.* Included both UK and US spelling and truncated terms for all the search terms listed.

Table A2

Full Search Strings

| Database | Search String | Number of Outcomes |
| --- | --- | --- |
| Ovid  APA PsycInfo [Coverage: 1806 to May Week 1 2024] | (digital peer support training) OR (digital peer support curriculum) OR (digital peer counselling) OR (digital peer disclosure) OR (digital peer reactions) OR (digital peer responses) OR (digital self-help group) OR (digital self help group) OR (digital support group))  AND ((randomized controlled trial on mental health) OR (intervention on mental health) OR (programs on mental health) OR (mental health) OR (wellbeing) OR (well-being) OR (psychological wellbeing) OR (psychological well-being) OR (randomized controlled trial on physical health) OR (intervention on physical health) OR (programs on physical health) OR (physical health) OR (physical wellbeing) OR (physical well-being)) ab, ti, sh, id.  (digital AND peer support) OR (digital AND peer support AND training) OR (digital AND peer support AND curriculum) OR (digital AND peer counselling) OR (digital AND peer disclosure) OR (digital AND peer reactions) OR (digital AND peer responses) OR (digital AND self-help group) OR (digital AND self help group) OR (digital AND support group))  AND ((randomized controlled trial on mental health) OR (intervention on mental health) OR (programs on mental health) OR (mental health) OR (wellbeing) OR (well-being) OR (psychological wellbeing) OR (psychological well-being) OR (randomized controlled trial on physical health) OR (intervention on physical health) OR (programs on physical health) OR (physical health) OR (physical wellbeing) OR (physical well-being)) ab, ti, sh, id.  ((digital OR eHealth OR web-based OR internet-based OR mobile OR gamification OR forums OR social media OR social networking sites OR Discord OR Facebook OR Instagram OR Reddit OR Snapchat OR Telegram OR TikTok OR Twitch OR Twitter OR WeChat OR WhatsApp OR YouTube OR online OR website OR app OR virtual OR video OR podcast OR interactive tutorial OR discussion board OR video games).  AND  (peer support OR peer support training OR peer support curriculum OR peer counselling OR peer disclosure OR peer reactions OR peer responses OR self-help group OR self help group OR support group)  AND  (mental health OR mental wellness OR mental well-being OR psychological well-being OR psychological functioning OR psychosocial functioning OR psychosocial well-being OR anxiety OR depression OR internalizing symptoms OR externalizing symptoms OR physical health OR weight management OR obesity OR lifestyle behaviour OR lifestyle promotion OR physical activity OR sleep OR dietary intake OR eating habits OR BMI OR blood pressure OR glucose level)) ab, ti, sh, id | 62,550 |
| Embase  (Embase.com)  [Coverage: 2013 to 2024] | (digital peer support: ab,ti,kw OR digital peer support training: ab,ti,kw OR digital peer support curriculum: ab,ti,kw OR digital peer counselling: ab,ti,kw OR digital peer disclosure: ab,ti,kw OR digital peer reactions: ab,ti,kw OR digital peer responses: ab,ti,kw OR digital self-help group: ab,ti,kw OR digital self help group: ab,ti,kw OR digital support group: ab,ti,kw)  AND (randomized controlled trial on mental health: ab,ti,kw OR intervention on mental health: ab,ti,kw OR programs on mental health: ab,ti,kw OR mental health: ab,ti,kw OR wellbeing: ab,ti,kw OR well-being: ab,ti,kw OR psychological wellbeing: ab,ti,kw OR psychological well-being: ab,ti,kw OR randomized controlled trial on physical health: ab,ti,kw OR intervention on physical health: ab,ti,kw OR programs on physical health: ab,ti,kw OR physical health: ab,ti,kw OR physical wellbeing: ab,ti,kw OR physical well-being: ab,ti,kw)  (digital:ab,ti,kw OR ehealth: ab,ti,kw OR web-based: ab,ti,kw OR internet-based: ab,ti,kw OR mobile: ab,ti,kw OR gamification: ab,ti,kw OR forums: ab,ti,kw OR Online: ab,ti,kw OR website: ab,ti,kw OR app: ab,ti,kw OR virtual: ab,ti,kw OR video: ab,ti,kw OR podcast: ab,ti,kw OR interactive tutorial: ab,ti,kw OR discussion board: ab,ti,kw OR video games: ab,ti,kw).  AND  (peer support: ab,ti,kw OR peer support training: ab,ti,kw OR peer support curriculum: ab,ti,kw OR peer counselling: ab,ti,kw OR peer disclosure: ab,ti,kw OR peer reactions: ab,ti,kw OR peer responses: ab,ti,kw OR self-help group: ab,ti,kw OR self help group: ab,ti,kw OR support group: ab,ti,kw)  AND  (mental health: ab,ti,kw OR mental wellness: ab,ti,kw OR mental well-being: ab,ti,kw OR psychological well-being: ab,ti,kw OR psychological functioning: ab,ti,kw OR psychosocial functioning: ab,ti,kw OR psychosocial well-being: ab,ti,kw OR anxiety: ab,ti,kw OR depression: ab,ti,kw OR internalizing symptoms: ab,ti,kw OR externalizing symptoms: ab,ti,kw OR mental health disorders: ab,ti,kw OR physical health: ab,ti,kw OR physical health: ab,ti,kw OR weight management: ab,ti,kw OR obesity: ab,ti,kw OR lifestyle behaviour: ab,ti,kw OR lifestyle promotion: ab,ti,kw OR physical activity: ab,ti,kw OR sleep: ab,ti,kw OR dietary intake: ab,ti,kw OR eating habits: ab,ti,kw OR BMI: ab,ti,kw OR blood pressure: ab,ti,kw OR glucose level: ab,ti,kw)  (social media: ab,ti,kw OR social networking sites: ab,ti,kw OR discord: ab,ti,kw OR facebook: ab,ti,kw OR instagram: ab,ti,kw OR reddit: ab,ti,kw OR snapchat: ab,ti,kw OR telegram: ab,ti.kw OR tiktok: ab,ti,kw OR twitch: ab,ti.kw OR twitter: ab,ti,kw OR wechat: ab,ti,kw OR whatsapp: ab,ti,kw OR youtube: ab,ti,kw)  AND  (peer support: ab,ti,kw OR peer support training: ab,ti,kw OR peer support curriculum: ab,ti,kw OR peer counselling: ab,ti,kw OR peer disclosure: ab,ti,kw OR peer reactions: ab,ti,kw OR peer responses: ab,ti,kw OR self-help group: ab,ti,kw OR self help group: ab,ti,kw OR support group: ab,ti,kw)  AND  (mental health: ab,ti,kw OR mental wellness: ab,ti,kw OR mental well-being: ab,ti,kw OR psychological well-being: ab,ti,kw OR psychological functioning: ab,ti,kw OR psychosocial functioning: ab,ti,kw OR psychosocial well-being: ab,ti,kw OR anxiety: ab,ti,kw OR depression: ab,ti,kw OR internalizing symptoms: ab,ti,kw OR externalizing symptoms: ab,ti,kw OR mental health disorders: ab,ti,kw OR physical health: ab,ti,kw OR weight management: ab,ti,kw OR obesity: ab,ti,kw OR lifestyle behaviour: ab,ti,kw OR lifestyle promotion: ab,ti,kw OR physical activity: ab,ti,kw OR sleep: ab,ti,kw OR dietary intake: ab,ti,kw OR eating habits: ab,ti,kw OR BMI: ab,ti,kw OR blood pressure: ab,ti,kw OR glucose level: ab,ti,kw)  [these search terms below do not apply to Embase]  (digital: ab,ti,kw AND peer support: ab,ti,kw AND training: ab,ti,kw OR digital: ab,ti,kw AND peer support: ab,ti,kw AND curriculum: ab,ti,kw OR digital: ab,ti,kw AND peer counselling: ab,ti,kw OR digital: ab,ti,kw AND peer disclosure: ab,ti,kw OR digital: ab,ti,kw AND peer reactions: ab,ti,kw OR digital: ab,ti,kw AND peer responses: ab,ti,kw OR digital: ab,ti,kw AND self-help group: ab,ti,kw OR digita: ab,ti,kw l AND self help group: ab,ti,kw OR digital: ab,ti,kw AND support group: ab,ti,kw)  AND (randomized controlled trial: ab,ti,kw AND mental health: ab,ti,kw OR mental health: ab,ti,kw AND intervention: ab,ti,kw OR mental health: ab,ti,kw AND programs: ab,ti,kw OR mental health: ab,ti,kw OR wellbeing: ab,ti,kw OR well-being: ab,ti,kw OR psychological wellbeing: ab,ti,kw OR psychological well-being: ab,ti,kw OR randomized controlled trial: ab,ti,kw AND physical health: ab,ti,kw OR intervention: ab,ti,kw AND physical health: ab,ti,kw OR programs: ab,ti,kw AND physical health: ab,ti,kw OR physical health: ab,ti,kw OR physical wellbeing: ab,ti,kw OR physical well-being: ab,ti,kw) | 19,811 |
| CINAHL complete  [Coverage: 1954 to 2024] | (TI (digital peer support OR digital peer support training OR digital peer support curriculum OR digital peer counselling OR digital peer disclosure OR digital peer reactions OR digital peer responses OR digital self-help group OR digital self help group OR digital support group) OR AB (digital peer support OR digital peer support training OR digital peer support curriculum OR digital peer counselling OR digital peer disclosure OR digital peer reactions OR digital peer responses OR digital self-help group OR digital self help group OR digital support group) OR MW (digital peer support OR digital peer support training OR digital peer support curriculum OR digital peer counselling OR digital peer disclosure OR digital peer reactions OR digital peer responses OR digital self-help group OR digital self help group OR digital support group)  AND (TI (randomized controlled trial on mental health OR intervention on mental health OR programs on mental health OR mental health OR wellbeing OR well-being OR psychological wellbeing OR psychological well-being OR randomized controlled trial on physical health OR intervention on physical health OR programs on physical health OR physical health OR physical wellbeing OR physical well-being) OR  AB (randomized controlled trial on mental health OR intervention on mental health OR programs on mental health OR mental health OR wellbeing OR well-being OR psychological wellbeing OR psychological well-being OR randomized controlled trial on physical health OR intervention on physical health OR programs on physical health OR physical health OR physical wellbeing OR physical well-being) OR  MW (randomized controlled trial on mental health OR intervention on mental health OR programs on mental health OR mental health OR wellbeing OR well-being OR psychological wellbeing OR psychological well-being OR randomized controlled trial on physical health OR intervention on physical health OR programs on physical health OR physical health OR physical wellbeing OR physical well-being))  (TI (digital AND peer support OR digital AND peer support AND training OR digital AND peer support AND curriculum OR digital AND peer counselling OR digital AND peer disclosure OR digital AND peer reactions OR digital AND peer responses OR digital AND self-help group OR digital AND self help group OR digital AND support group) OR  AB (digital AND peer support OR digital AND peer support AND training OR digital AND peer support AND curriculum OR digital AND peer counselling OR digital AND peer disclosure OR digital AND peer reactions OR digital AND peer responses OR digital AND self-help group OR digital AND self help group OR digital AND support group) OR  MW (digital AND peer support OR digital AND peer support AND training OR digital AND peer support AND curriculum OR digital AND peer counselling OR digital AND peer disclosure OR digital AND peer reactions OR digital AND peer responses OR digital AND self-help group OR digital AND self help group)OR digital AND support group))  AND (TI (randomized controlled trial on mental health) OR (mental health AND intervention) OR (mental health AND programs) OR (mental health) OR (wellbeing) OR (well-being) OR (psychological wellbeing) OR (psychological well-being) OR (randomized controlled trial on physical health) OR (intervention on physical health) OR (programs on physical health) OR (physical health) OR (physical wellbeing) OR (physical well-being) OR  AB (randomized controlled trial on mental health) OR (mental health AND intervention) OR (mental health AND programs) OR (mental health) OR (wellbeing) OR (well-being) OR (psychological wellbeing) OR (psychological well-being) OR (randomized controlled trial on physical health) OR (intervention on physical health) OR (programs on physical health) OR (physical health) OR (physical wellbeing) OR (physical well-being) OR  MW (randomized controlled trial on mental health) OR (mental health AND intervention) OR (mental health AND programs) OR (mental health) OR (wellbeing) OR (well-being) OR (psychological wellbeing) OR (psychological well-being) OR (randomized controlled trial on physical health) OR (intervention on physical health) OR (programs on physical health) OR (physical health) OR (physical wellbeing) OR (physical well-being)  (TI (digital OR eHealth OR web-based or internet-based OR mobile OR gamification OR forums OR social media OR social networking sites OR Discord OR Facebook OR Instagram OR Reddit OR Snapchat OR Telegram OR TikTok OR Twitch OR Twitter OR WeChat OR WhatsApp OR YouTube OR online OR website OR app OR virtual OR video OR podcast OR interactive tutorial OR discussion board OR video games) OR  AB (digital OR eHealth OR web-based OR internet-based OR mobile OR gamification OR forums OR social media OR social networking sites OR Discord OR Facebook OR Instagram OR Reddit OR Snapchat OR Telegram OR TikTok OR Twitch OR Twitter OR WeChat OR WhatsApp OR YouTube OR online OR website OR app OR virtual OR video OR podcast OR interactive tutorial OR discussion board OR video games) OR  MW (digital OR eHealth OR web-based OR internet-based OR mobile OR gamification OR forums OR social media OR social networking sites OR Discord OR Facebook OR Instagram OR Reddit OR Snapchat OR Telegram OR TikTok OR Twitch OR Twitter OR WeChat OR WhatsApp OR YouTube OR online OR website OR app OR virtual OR video OR podcast OR interactive tutorial OR discussion board OR video games))  AND  (TI(peer support or peer support training OR peer support curriculum OR peer counselling OR peer disclosure OR peer reactions OR peer responses OR self-help group OR self help group OR support group) OR  AB (peer support or peer support training OR peer support curriculum OR peer counselling OR peer disclosure OR peer reactions OR peer responses OR self-help group OR self help group OR support group) OR  MW (peer support or peer support training OR peer support curriculum OR peer counselling OR peer disclosure OR peer reactions OR peer responses OR self-help group OR self help group OR support group)  AND  (TI (mental health OR mental wellness OR mental well-being OR psychological well-being OR psychological functioning OR psychosocial functioning OR psychosocial well-being OR anxiety OR depression OR internalizing symptoms OR externalizing symptoms OR mental health disorders OR randomized controlled trial on physical health OR intervention on physical health OR programs on physical health OR physical health OR physical wellbeing OR physical well-being) OR  AB (mental health OR mental wellness OR mental well-being OR psychological well-being OR psychological functioning OR psychosocial functioning OR psychosocial well-being OR anxiety OR depression OR internalizing symptoms OR externalizing symptoms OR mental health disorders OR randomized controlled trial on physical health OR intervention on physical health OR programs on physical health OR physical health OR physical wellbeing OR physical well-being) OR  MW (mental health OR mental wellness OR mental well-being OR psychological well-being OR psychological functioning OR psychosocial functioning OR psychosocial well-being OR anxiety OR depression OR internalizing symptoms OR externalizing symptoms OR mental health disorders OR randomized controlled trial on physical health OR intervention on physical health OR programs on physical health OR physical health OR physical wellbeing OR physical well-being)) | 12,082 |
| Total | | 94,443 |

Table A3

Characteristics of Digital Peer Support Interventions and Mental Health

|  | Control | | | Digital Peer Support | | | Alternative Treatment | | |  |  |
| --- | --- | --- | --- | --- | --- | --- | --- | --- | --- | --- | --- |
| **Study** | **M** | **SD** | **Total** | **M** | **SD** | **Total** | **M** | **SD** | **Total** | **Weight** | **Standardized Mean Difference** |
| Bilich et al. 2008 | 16.98 | 7.74 | 34 | 11.10 | 7.44 | 16 | 14.40 | 9.39 | 23 | 0.851 | 0.775^a^, 0.390^b^ |
|  | (17.22) | (6.35) | - | (17.78) | (5.65) | - | (17.57) | (7.08) | - | - | 0.093^a^, 0.033^b^ |
| Buglione et al. 1990 | - | - | - | 23.47 | 6.27 | 17 | 22.88 | 8.14 | 16 | 0.794 | 0.081^b^ |
|  | - | - | - | (33.22) | (6.46) | - | (32.18) | (7.04) | - | - | 0.154^b^ |
| Buntrock et al. 2016 | 19.42 | 6.85 | 204 | 16.84 | 5.24 | 202 | - | - | - | 0.877 | 0.423^a^ |
|  | (26.42) | (7.99) | - | (26.25) | (7.85) | - | - | - | - | - | 0.021^a^ |
| Ehlers et al. 2003 | 12.30 | 9.30 | 27 | 14.60 | 7.75 | 25 | 6.15 | 5.95 | 28 | 0.847 | 0.269^a^, 1.223^b^ |
|  | (20.20) | (7.30) | - | (22.55) | (9.55) | - | (23.55) | (8.15) | - | - | 0.276^a^, 0.113^b^ |
| Geraedts et al. 2014 | 13.30 | 6.35 | 171 | 11.70 | 7.20 | 171 | - | - | - | 0.871 | 0.236^a^ |
|  | (18.15) | (5.10) | (231) | (18.15) | (5.65) | (231) | - | - | - | - | 0.000^a^ |
| Horgan et al. 2013 | - | - | - | 33.50 | - | 118 | - | - | - | 0.859 | 0.097^c^ |
|  | - | - | - | (37.00) | (7.94) | (118) | - | - | - | - | - |
| Leow et al. 2015 | 4.43 | 5.00 | 42 | 1.58 | 2.22 | 38 | - | - | - | 0.847 | 0.737^a^ |
|  | (3.40) | (3.96) | - | (3.44) | (3.56) | - | - | - | - | - | 0.011^a^ |
| McKay et al. 2002 | 15.31 | 9.15 | 33 | 13.86 | 12.62 | 30 | 15.65 | 11.34 | 70 | 0.862 | 0.132^a^, 0.149^b^ |
|  | (15.27) | (12.25) | - | (15.83) | (12.79) | - | (17.36) | (11.34) | - | - | 0.045^a^, 0.127^b^ |
| Moir 2016 | 4.85 | 3.44 | 121 | 4.65 | 3.69 | 111 | - | - | - |  | 0.056^a^ |
|  | (4.78) | (3.61) | 142 | (4.99) | (3.65) | 133 | - | - | - | - | 0.058^a^ |
| Titov et al. 2008 | 27.73 | 10.14 | 49 | 20.44 | 8.18 | 50 | - | - | - | 0.854 | 0.791^a^ |
|  | (29.93) | (10.22) | - | (29.27) | (9.13) | - | - | - | - | - | 0.068^a^ |
| Titov et al. 2008 | 28.62 | 8.90 | 40 | 19.87 | 9.43 | 41 | - | - | - | 0.848 | 0.954^a^ |
|  | (0.84) | (8.50) | - | (30.16) | (10.36) | - | - | - | - | - | 3.094^a^ |
| Travis et al. 2010 | - | - | - | 24.50 | 19.00 | 32 | - | - | - | 0.792 | 0.099^c^ |
|  | - | - | - | (26.00) | (10.10) | 54 | - | - | - | - | - |
| Pavarini et al. 2023 | 45.04 | 9.28 | 50 | 55.56 | 6.3 | 50 | - | - | - | 0.855 | 0.939^c^,0.932^a^ |
|  | (46.84) | (6.22) | - | (46.48) | (6.68) | - | - | - | - | - | - |
| Donovan et al. 2019 | - | - | - | 6.6 | 3.8 | 17 | - | - | - | 0.711 | 0.144^c^ |
|  | - | - | - | (6.1) | (3.1) | - | - | - | - | - | - |
| Marinova et al. 2022 | - | - | - | 17.85 | 5.79 | 606 | - | - | - | 0.880 | 0.057^c^ |
|  | - | - | - | (18.2) | (6.43) | - | - | - | - | - | - |
| Gregoire et al. 2022 | 14.09 | 6.69 | 53 | 11.26 | 6.9 | 54 | - | - | - | 0.856 | 0.30^c^, 0.416^a^ |
|  | (13.75) | (6.61) | - | (13.39) | (6.75) | - | - | - | - | - | - |
| Suffoleto et al. 2021 | - | - | - | 1.36 | 0.96 | 26 | 1.57 | 1 | 26 | 0.827 | 0.073^a^,0.214^b^ |
|  | - | - | - | (1.43) | (0.95) | - | (1.81) | (0.9) | - | - | - |
| Sin et al. 2022 | 43.3 | 9.19 | 204 | 44.5 | 8.31 | 203 | - | - | - | 0.877 | 0.137^a^ |
| Baumel et al. 2018 | - | - | - | 19.18 | 9.23 | 19 | - | - | - | 0.729 | 0.975^c^ |
|  | - | - | - | (36.11) | (13.34) | - | - | - | - | - | - |
| Birrell et al. 2023 | - | - | - | 7.73 | 5.55 | 83 | 7.21 | 5.64 | 83 | 0.866 | 0.059^c^,0.093^b^ |
|  | - | - | - | (8.06) | (5.66) | - | (6.14) | (5.31) | - | - | - |
| Fortuna et al. 2022 | 34.77 | 6.63 | 10 | 32.11 | 1.99 | 11 | - | - | - | 0.744 | 0.543 |
| Klimczak et al. 2023 | 45.4 | 17 | 77 | 33.8 | 24.4 | 76 | 33.6 | 22.2 | 77 | 0.871 | 0.453^c^,0.552^a^,  0.009^b^ |
|  | 50.8 | (27) | - | (45.8) | (28.4) | - | (49) | (25.1) | - | - | - |
| Wright et al. 2022 | - | - | - | 40.1 | 8.7 | 40 | - | - | - | 0.810 | 0.80^c^ |
|  | - | - | - | (47.1) | (6.6) | - | - | - | - | - | - |
| Drysdale et al. 2021 | 12.43 | 2.06 | 30 | 15.13 | 1.96 | 30 | 15.92 | 2.66 | 30 | 0.851 | 0.952^c^,0.338^b^,  0.934^a^ |
|  | (12.85) | (3.25) | - | (12.8) | (0.92) | - | (12.8) | (1.74) | - | - | - |
| Pauksztat et al. 2022 | 1.62 | 0.18 | 66 | 1.30 | 0.18 | 65 | - | - | - | 0.862 | 0.978^c^ |
| Kaplan 2011 | 2.02 | 0.06 | 150 | 1.88 | 0.04 | 150 | - | - | - | - | 0.920^c^, 0.975^a^ |
|  | (2.05) | (0.06) | - | (1.96) | (0.04) | - | - | - | - | - | - |
| Tomasino et al. 2017 | 8.2 | 5.7 | 15 | 6.4 | 4.2 | 16 | 5.1 | 2.8 | 16 | 0.821 | 0.992^c^,  0.360^a^,0.364^b^ |
|  | (9.3) | (3.7) | - | (11.2) | (5.4) | - | (10.6) | (3.2) | - | - | - |
| Schulz et al. 2016 | 14.41 | 11.42 | 49 | 10.27 | 9.87 | 50 | 10.35 | 10.22 | 50 | 0.864 | 0.748^c^,  0.388^a^,0.008^b^ |
|  | (17.97) | (11.59) | - | (17.88) | (10.46) | - | (19.43) | (10.22) | - | - | - |
| Hensel et al. 2019 | - | - | - | 11.5 | 6.4 | 727 | 14.2 | 6.8 | 728 | 0.882 | 0.496^c^,0.409^b^ |
|  | - | - | - | (14.8) | (6.9) | - | (16) | (6.5) | - | - | - |
| Nosek et al. 2016 | - | - | - | 7.81 | 3.38 | 19 | - | - | - | 0.729 | 0.74^c^ |
|  | - | - | - | (11.4) | (6.36) | - | - | - | - | - | - |
| Smith et al. 2011 | - | - | - | 4 | 3 | 25 | 11.1 | 13.6 | 25 | 0.875 | 0.809^c^,0.721^b^ |
|  | - | - | - | (9.1) | (8.4) | - | (3.5) | (2.8) | - | - | - |
| Setoyama et al. 2011 | 13.4 | 8.7 | 465 | 12.5 | 6.9 | 465 | - | - | - | 0.878 | 0.102^a^ |
| Lepore et al. 2019 | - | - | - | 5.94 | 0.34 | 183 | - | - | - | 0.868 | 0.918^c^ |
|  | - | - | - | (6.93) | (0.28) | - | - | - | - | - | - |
| Pretorius et al. 2009 | - | - | - | 2.9 | 0.2 | 101 | - | - | - | 0.855 | 0.932^c^ |
|  | - | - | - | (3.9) | (0.1) | - | - | - | - | - | - |
| McKay et al. 2001 | 19.9 | 14.2 | 34 | 14.9 | 12.5 | 34 | - | - | - | 0.846 | 0.166^c^,0.374^a^ |
|  | (17.6) | (10.4) | - | (16.9) | (11.6) | - | - | - | - | - | - |
| Freeman et al. 2008 | - | - | - | 0.91 | 0.47 | 117 | 1.04 | 0.64 | 117 | 0.872 | 0.408^c^,0.232^b^ |
|  | - | - | - | (1.13) | (0.6) | - | (1.31) | (0.64) | - | - | - |
| Wikerson et al. 2018 | - | - | - | 20 | - | 12 | - | - | - | 0.642 | 0.62^c^ |
|  | - | - | - | (29.5) | - | 12 | - | - | - | - | - |
| Houston et al. 2002 | - | - | - | - | - | - | - | - | - | 0.855 | 2.7 (relative odd-ratio)^a^ |
| Mouthan et al. 2013 | 4.1 | 3.5 | 150 | 3.6 | 3.2 | 150 | - | - | - | 0.874 | 0.027^c^,0.149^a^ |
|  | (4.13) | (4.26) | - | (3.69) | (3.5) | - | - | - | - | - | - |
| Nelson et al. 2014 | - | - | - | 18.21 | 13.2 | 19 | - | - | - | 0.729 | 0.9680.299^c^ |
|  | - | - | - | (21.86) | (11.11) | - | - | - | - | - | - |
| Stevens et al. 2022 | - | - | - | 24.1 | 6.1 | 151 | 24.6 | 6.1 | 151 | 0.875 | 0.475^c^,0.082^b^ |
|  | - | - | - | (27) | (6.1) | - | - | - | - | - | - |
| Yeung et al. 2021 | - | - | - | 4.2 | 3.1 | 253 | - | - | - | 0.873 | 0.209^c^ |
|  | - | - | - | (6.5) | (3.6) | - | - | - | - | - | - |
| Houwen et al. 2010 | 18.5 | 13.3 | 126 | 20.6 | 13.6 | 127 | - | - | - | 0.873 | 0.162^c^,0.156^a^ |
|  | (20.9) | (12) | - | (22.8) | (13.4) | - | - | - | - | - | - |
| Bautista et al. 2022 | 10.92 | 5.15 | 17 | 7.5 | 10.12 | 18 | - | - | - | 0.799 | 0.622^c^,0.426^a^ |
|  | (14) | (5) | - | (12.4) | (4.65) | - | - | - | - | - | - |
| Joyce et al. 2018 | - | - | - | 27.5 | 4.9 | 29 | - | - | - | 0.782 | 0.288^c^ |
|  | - | - | - | (26) | (5.5) | - | - | - | - | - | - |
| Linke 2007 | - | - | - | 1 | 0.89 | 10000 | - | - | - | 0.884 | 0.778^c^ |
|  | - | - | - | 1.70 | (0.91) | - | - | - | - | - | - |
| Paulson & Casile 2015 | - | - | - | 24.16 | 18.84 | 6 | - | - | - | 0.414 | 0.340^c^ |
|  | - | - | - | (18.5) | (14.06) | - | - | - | - | - | - |
| Kramer et al. 2015 | - | - | - | 21.35 | 11.77 | 270 | - | - | - | 0.873 | 0.187^c^ |
|  | - | - | - | (23.59) | (12.2) | - | - | - | - | - | - |
| Ebert et al. 2013 | 0.83 | 0.66 | 200 | 0.83 | 0.64 | 200 | - | - | - | 0.877 | 0.968^c^,0.00^a^ |
|  | - | - | - | (1.5) | (0.69) | - | - | - | - | - | - |
| Owen et al. 2016 | - | - | - | - | - | - | - | - | - | 0.874 | 1.01 odd ratio^c^ |
| Niemiec et al. 2018 | - | - | - | 3.5  (4.7) | 1.4  (1.2) | 37 | - | - | - | 0.804 | 0.920^c^ |
| Zheng et al. 2021 | 0.07mean change | 0.05 | 477 | 0.08mean change | 0.10 | 477 | - | - | - | 0.881 | 0.128^c^ |
| Houston et al. 2002 | - | - | - | - | - | 103 | - | - | - | 0.855 | 0.597^c^ |
| Evette et al. 2007 | 1.64 | 0.52 | 52 | 1.63 | 0.68 | 26 | 1.72 | 0.56 | 26 | 0.856 | 0.017^a^, 0.144^b^ |
| O’Dea et al. 2020 | 10.02 | 4.77 | 95 | 10.49 | 4.87 | 98 | - | - | - | 0.869 | 0.098^a^ |
|  | (10.70) | (4.73) | - | (11.55) | (4.72) | - | - | - | - | - | - |
| Andersson et al. 2005 | 18.77 | 8.37 | 49 | 13.00 | 7.83 | 36 | - | - | - | 0.849 | 0.712^a^ |
|  | 20.03) | (8.07) | - | (19.23) | (6.87) | - | - | - | - | - | - |
| Lieberman & Goldstein 2005 | - | - | - | 15.30 | 1.20 | 91 | - | - | - | 0.852 | 4.520^c^ |
|  | - | - | - | (20.00) | (0.85) | - | - | - | - | - | - |
| Lee Dennis 2003 | 15.20 | 4.85 | 22 | 13 | 3.96 | 20 | - | - | - | 0.810 | 0.497^a^ |
|  | (16.29) | (4.47) | - | (15.93) | (4.06) | - | - | - | - | - | 0.084^a^ |
| Goldberg et al. 2015 | - | - | - | 2.24 | 1.43 | 2817 | - | - | - | 0.883 | 0.18prei-posti |
|  | - | - | - | (2.55) | (1.45) | - | - | - | - | - | - |
| Imanaka et al. 2013 | - | - | - | 0.5mean change | 17.1 | 96 | 1.1mean change | 11.1 | 97 | 0.869 | 0.698prei-posti |
| Kelly et al. 2021 | - | - | - | 3.96mean change | 8.32 | 72 | - | - | - | 0.843 | 0.410prei-posti |
| Morriss et al. 2021 | 3.26mean change | 1.89 | 395 | 3.62mean change | 2.12 | 695 | - | - | - | 0.881 | 0.295prei-posti |
| Shorey et al. 2019 | 12.4mean change | 2.1 | 69 | 11.4mean change | 2 | 69 | - | - | - | 0.863 | 0.96prei-posti |
| Bratava et al. 2023 | - | - | - | 6.47 | 1.65 | 815 | - | - | - | 0.881 | 0.569prei-posti |
|  | - | - | - | (7.41) | (1.65) | - | - | - | - | - | - |
| Dennis et al. 2009 | 34.4 | 12.07 | 350 | 33.63 | 11.01 | 351 | - | - | - | 0.880 | 0.129prei-posti |
|  | (36.88) | (12.84) | - | (35.1) | (11.85) | - | - | - | - | - | 0.067postc-posti |
| Baustita et al. 2022 | 10.29 | 5.15 | 18 | 7.5 | 3.78 | 17 | - | - | - | 0.799 | 0.082prei-posti |
|  | (11.4) | (5) | - | (12.4) | (4.65) | - | - | - | - | - | 0.763postc-posti |
| Kruzan et al. 2022 | 1.62mean change | 0.18 | 66 | 1.3mean change | 0.18 | 65 | - | - | - | 0.862 | 0.479prei-posti |
| Gillard et al. 2022 | - | - | 295 | - | - | 295 | - | - | - | 0.879 | 0.040postc-posti |
| Summers et al. 2021 | - | - | - | 4.74 | 3.82 | 347 | - | - | - | 0.876 | 0.550prei-posti |
|  | - | - | - | (7.07) | (4.62) | - | - | - | - | - | - |
| Kahl et al. 2020 | - | - | - | 17.7 | 12.74 | 1982 | - | - | - | 0.883 | 0.387prei-posti |
|  | - | - | - | (22.55) | 12.3 | - | - | - | - | - | - |
| Economides et al. 2019 | - | - | - | - | - | 102 | - | - | - | 0.855 | 0.454prei-posti |
| Moir et al. 2016 | 5.02 | 3.19 | 137 | 5.15 | 3.72 | 138 | - | - | - | 0.874 | 0.122prei-posti |
|  | (4.82) | (3.67) | - | (5.61) | (3.85) | - | - | - | - | - | 0.038postc-posti |

*Note.* Pre-scores, where available, are added in parentheses. The mean (M), standard deviation (SD), and number of participants (Total) are provided according to their group (control, digital peer support, alternative treatment). Standardized Mean Differences (SMD) are provided, where a = SMD between digital peer support and control groups; b = SMD between digital peer support and alternative digital treatment groups, and c = SMD between pre- and post-digital peer support intervention. Odds ratios were converted to Cohen’s d/SMD in analyses.

Table A4

Characteristics of Digital Peer Support Interventions and Physical Health

|  | Control | | | Digital Peer Support | | | Alternative Treatment | | |  |  |
| --- | --- | --- | --- | --- | --- | --- | --- | --- | --- | --- | --- |
| **Study** | **M** | **SD** | **Total** | **M** | **SD** | **Total** | **M** | **SD** | **Total** | **Weight** | **Standardized Mean Difference (SMD)** |
| DeBar et al. 2009 | - | - | - |  |  | 228 | - | - | - | 1.404 | 0.105^a^ |
| Goldberg et al. 2015 | - | - | - | 116.53  (118.96) | 14.08  (15.13) | 2817 | - | - | - | 1.466 | 0.166^c^ |
| Mamede et al. 2021 | 10618.6 | 4377.3 | 149 | 10901.8 | 5068.3 | 149 | - | - | - | 1.419 | 0.060^a^ |
| Keyseling et al. 2008 | 5.7 | 0.1 | 118 | 5.8 | 0.1 | 118 | - | - | - | 1.406 | 0.091^a^ |
| Lara et al. 2016 | 26.8  (26.6) | 2.54  (2.52) | 37 | 26.2  (27.3) | 2.52  (2.54) | 38 | - | - | - | 1.280 | 0.435^c^  0.079^a^ |
| Moravcova et al. 2022 | 111.6  (117.6) | 23.2  (22.3) | 50 | 107.8  (114.30) | 19  (20) | 50 | - | - | - | 1.324 | 0.333^c^  0.179^a^ |
| Tsai & Liu 2015 | 522.1  (519.1) | 101.1  (114.2) | 57.5 | 556  (526.5) | 115.7  (128) | 58 | - | - | - | 1.331 | 0.236^c^  0.312^a^ |
| Mi et al. 2022 | - | - | - | 0.69  (1.25) | 2.56  (3.77) | 268 | - | - | - | 1.414 | 0.174^c^ |
| Sharps et al. 2019 | 1.38  (1.36) | 0.33  (0.31) | 10 | 1.28  (1.47) | 0.27  (0.28) | 10 | - | - | - | 0.901 | 0.690^c^  0.385^a^ |
| West et al. 2016 | 7.1  (8.3) | 3.6  (3.6) | 29 | 9.9  (7.8) | 3.1  (4.5) | 29 | - | - | - | 1.231 | 0.54^c^  0.834^a^ |
| Watanabe-Ito et al. 2020 | - | - | - | 6.2  (4.6) | 5.7  (2.5) | 42 | - | - | - | 1.153 | 0.364^c^ |
| Carlsen et al. 2013 | 0.54  (0.21) | 0.88  (1.14) | 113 | 0.59  (0.14) | 0.87  (1.13) | 113 | - | - | - | 1.404 | 0.446^c^  0.057^a^ |
| Morgan et al. 2011 | 3mean change | 3.45 | 32.5 | 4.8 mean change | 4.35 | 33 | - | - | - | 1.254 | 0.053^c^ |
| Leahey et al. 2016 | 4 | 6.3 | 25 | 8.6 | 6.3 | 25 | 9.7 | 7.1 | 25 | 1.280 | 0.164^b^  0.730^a^ |
| Tate et al. 2006 | 1544.2  1869.7 | 651.7  (778.9) | 64 | 1468.2  (2042.6) | 449.1  (875.6) | 64 | 1381.7  (1911.6) | 448.2  (770.9) | 64 | 1.392 | 0.825^c^  0.193^b^  0.136^a^ |
| Harvey-Berino et al. 2004 | - | - | - | 7.6 | 7.3 | 127 | 6.5 | 5.5 | 128 | 1.411 | 0.170^b^ |
| Lim et al. 2020 | - | - | - | 2.6 mean difference | 1.3 | 108 | - | - | - | 1.334 | 0.382^a^ |
| Ross et al. 2022 | - | - | - | 83.3  (87.8) | 5.1  (5.1) | 1811 | 85.8  (86.3) | 2.9  (2.2) | 1812 | 1.467 | 0.602^b^ |
| Fiks et al. 2017 | - | - | - | - | - | 87 | - | - | - | 1.304 | 0.450^c^ |
| Tate el al. 2001 | 4.1 mean change | 4.5 | 45 | 1.6 mean change | 3.3 | 46 | - | - | - | 1.311 | 0.400^c^ |
| Pretlow et al. 2015 | - | - | - | - | - | 43 | - | - | - | 1.160 | 0.700^c^ |
| Webber et al. 2008 | - | - | - | 3.71 mean change | 4.46 | 21 | 5.22 mean change | 4.72 | 22 | 1.257 | 0.102^c^ |
| Pappa et al. 2017 | - | - | - | - | - | 107886 | - | - | - | 1.471 | 0.830^c^ |
| Anderson 2022 | 218.40  (218.44) | 40.37  (43.25) | 26 | 212.54  (218.75) | 58.15  (49.38) | 27 | 215.03  (219.95) | 43.36  (45.89) | 27 | 1.291 | 0.117^c^  0.049^b^ |
| Johnson & Wardle 2011 | - | - | 1810 | - | - | 1811 | - | - | - | 1.467 | 0.043^a^ |
| Turner-McGrievy et al. 2013 | - | - | - | - | - | 23 | - | - | 24 | 1.182 | 0.480^a^ |
| Lee et al. 2018 | - | - | - | 32.5  (33.1) | 4.7  (4.8) | 22 | - | - | - | 0.940 | 0.126^c^ |
| Dennison et al. 2014 | 91.34  (91.4) | 20.15  (20.31) | 262 | 89.59  (91.86) | 20.65  (20.96) | 262 | 90  (92.02) | 19.89  (20.09) | 262 | 1.451 | 0.086^a^  0.020^b^ |
| Hageman et al. 2017 | 201.7  (196.8) | 33.5  (36.8) | 100 | 210.5  (200.4) | 35.8  (37.4) | 100 | 196.6  (200.4) | 40.8  (43.7) | 101 | 1.420 | 0.006^a^  0.128^b^ |
| Richardson et al. 2010 | 5428  (3859) | 2667  (1586) | 162 | 6575  (4601) | 3127  (2074) | 162 | - | - | - | 1.423 | 0.388^a^ |
| An et al. 2013 | 2.64mean change | 2.2 | 566 | 7.58 mean change | 7.10 | 566 | 6.51 mean change | 6.04 | 566 | 1.462 | 0.036^c^ |
| Rosas et al. 2022 | 3.3mean change | 6.0 | 66 | 3.8mean change | 8.4 | 66 | 0.09mean change | 4.6 | 68 | 1.395 | 0.438^c^ |
| Li et al. 2021 | - | - | - | - | - | 353 | - | - | - | 1.428 | 0.386^c^ |
| Imanaka et al. 2013 | - | - | - | 0.61mean change | 1.0 | 96 | 0.3mean change | 0.8 | 97 | 1.392 | 0.282^c^ |
| Sepah et al. 2017 | - | - | - | 22.7mean change | 0.70 | 220 | - | - | - | 1.402 | 0.691^c^ |
| Ali et al. 2021 | - | - | - | 6.41  (6.33) | 1.18  (1.77) | 79 | - | - | - | 1.289 | 0.073^c^ |
| Travis et al. 2010 | - | - | - | 38.7  (42.8) | 13.9  (17.7) | 54 | - | - | - | 1.215 | 0.258^c^ |
| Hensel et al. 2019 | 0.69  (0.68) | 0.15  (0.16) | 406 | 0.71  (0.68) | 0.17  (0.16 | 406 | - | - | - | 1.452 | 0.182^c^  0.125^a^ |
| Dennis et al. 2009 | 2.86  (4.85) | 1.62  (1.52) | 350 | 2.83  (4.97) | 1.53  (1.62) | 351 | - | - | - | 1.449 | 0.165^c^  0.019^a^ |
| Linke et al. 2007 | - | - | - | 0.87  (1.32) | 0.69  (0.73) | 8933 | - | - | - | 1.470 | 0.634^c^ |
| Owen et al. 2016 | - | - | 149 | - | - | 150 | - | - | - | 1.420 | 0.496^a^ |
| Stevens et al. 2022 | - | - | - | 1.0mean change | 1.3 | 151 | 0.1 mean change | 1.3 | 151 | 1.420 | 0.769^c^ |
| Paulson et al. 2015 | - | - | - | 24.16  (18.5) | 18.81  (14.06) | 6 | - | - | - | 0.321 | 0.341^c^ |
| Freeman et al. 2008 | 1.04  (1.31) | 0.64  (0.64) | 119 | 0.91  (1.13) | 0.47  (0.60) | 119 | - | - | - | 1.407 | 0.408^c^  0.232^a^ |
| McKeon et al. 2021 | - | - | - | 43.6  (48) | 9.5  (7.9) | 166 | - | - | - | 0.973 | 0.504^c^ |
| Suffoleto et al. 2021 | 1.61  (1.51) | 1.11  (1.06) | 26 | 1.22  (1.37) | 0.03  (0.89) | 26 | - | - |  | 1.207 | 0.283^c^  0.355^a^ |

*Note.* Pre-scores, where available, are added in parentheses. The mean (M), standard deviation (SD), and number of participants (Total) are provided according to their group (control, digital peer support, alternative treatment). Standardized Mean Differences (SMD) are provided, where a = SMD between digital peer support and control groups; b = SMD between digital peer support and alternative digital treatment groups, and c = SMD between pre- and post-digital peer support intervention. Odds ratios were converted to Cohen’s d/SMD in analyses.

Table A5

Characteristics of Digital Peer Support Interventions and Mental Health

| **Authors** | **Year** | **N** | **SMD** | **Mean**  **age** | **Gender** | **Country** | **Ethnic composition** | **Plat form** | **Uptake (100%)** | **Duration** | **Follow up assessment** | **Study design** | **Source of support** |
| --- | --- | --- | --- | --- | --- | --- | --- | --- | --- | --- | --- | --- | --- |
| Leow et al. | 2015 | 80 | 0.8440 | 47.16 | 67.50 | 2 | 85% Chinese, 10% Malay, 3.8% Indian, 1.3% Caucasian | 2 | 54.80 | 16 | 16 | 4 | 3 |
| Ludman et al. | 2007 | 104 | 0.0756 | 50.20 | 71.00 | 1 | 87% Caucasian | 6 | 83.00 | 48 | 12 | 4 | 3 |
| O'Dea et al. | 2020 | 193 | 0.0925 | 14.82 | 86.53 | 1 | 97% Australian, 3% Aboriginal and/or Torres Strait Islander | 1 | 68.90 | 4 | 12 | 3 | 2 |
| O'Dea et al. | 2020 | 193 | 0.1330 | 14.82 | 86.53 | 1 | 97% Australian, 3% Aboriginal and/or Torres Strait Islander | 1 | 68.90 | 4 | 12 | 3 | 2 |
| Ehlers et al. | 2003 | 80 | 0.6790 | - | - | 1 | - | 6 | 92.90 | 12 | 36 | 4 | 3 |
| Ehlers et al. | 2003 | 80 | 0.7390 | - | - | 1 | - | 6 | 92.90 | 12 | 36 | 4 | 3 |
| Horgan et al. | 2013 | 118 | 0.0970 | 20.60 | 35.60 | 1 | 98% White | 2 | 13.60 | 12 | - | 1 | 1 |
| Buntrock et al. | 2016 | 406 | 0.3000 | 45.00 | 73.90 | 1 | 83.5% White, 0.2% African American, 0.2% Hispanic, 16% Not reported | 4 | 64.40 | 48 | 12 | 3 | 3 |
| Bilich et al. | 2008 | 89 | 0.3630 | - | 66.90 | 1 | - | 6 | 76.20 | 8 | 4 | 3 | 3 |
| Bilich et al. | 2008 | 89 | 0.5540 | - | 66.90 | 1 | - | 6 | 76.20 | 8 | 4 | 3 | 3 |
| McKay et al. | 2002 | 133 | 0.1030 | 59.00 | 53.10 | 1 | - | 2 | 84.00 | 12 | - | 4 | 2 |
| Andersson et al. | 2005 | 85 | 0.6260 | 36.40 | 78.00 | 1 | - | 4 | 62.40 | 10 | 24 | 3 | 2 |
| Andersson et al. | 2005 | 85 | 0.0960 | 36.40 | 78.00 | 1 | - | 4 | 62.40 | 10 | 24 | 3 | 2 |
| Geraedts et al. | 2014 | 231 | 0.1990 | 43.40 | 62.30 | 1 | 95.2% Netherlands, 4.8% other | 4 | 51.70 | 8 | 48 | 3 | 3 |
| Geraedts et al. | 2014 | 231 | 0.1440 | 43.40 | 62.30 | 1 | 95.2% Netherlands, 4.8% other | 4 | 51.70 | 8 | 48 | 3 | 3 |
| Lieberman & Goldstein | 2005 | 91 | 0.910 | 46.20 | 100.00 | 1 | - | 2 | 80.00 | 24 | - | 1 | 1 |
| Titov et al. | 2008 | 99 | 0.7000 | 38.13 | 59.00 | 1 | - | 4 | 78.00 | 10 | - | 3 | 1 |
| Titov et al. | 2008 | 81 | 0.9020 | 36.79 | 63.00 | 1 | - | 4 | 78.00 | 10 | - | 3 | 1 |
| Travis et al. | 2010 | 32 | 0.2740 | 55.40 | 25.00 | 1 | - | 6 | 59.30 | 12 | - | 1 | 2 |
| Lee Dennis | 2003 | 40 | 0.5520 | - | 100.00 | 1 | born in Canada 85% | 6 | 100.00 | 1 | 4 | 3 | 2 |
| Buglione et al. | 1990 | 33 | 0.3450 | - | 42.40 | 1 | - | 4 | 73.00 | 6 | - | 4 | 3 |
| Pavarini et al. | 2023 | 100 | 0.9390 | 16-17 | 84.00 | 1 | 42% White British, 18% White Irish, 14% Black British, 2% Mixed | 5 | 78.00 | 1 | 3 | 3 | 1 |
| Donovan et al. | 2019 | 17 | 0.1440 | 19.30 | 50.00 | 1 | 67% White, 10% African American, 5% Asian, 14% Multiracial, 5% unknown | 1 | 88.20 | 4 | - | 1 | 1 |
| Marinova et al. | 2022 | 606 | 0.0570 | 17.30 | 78.70 | 1 | - | 4 | 40.00 | 12 | - | 1 | 1 |
| Gregoire et al. | 2022 | 107 | 0.3000 | 26.00 | 76.60 | 1 | 87% Canadian | 5 | 49.00 | 20 | 12 | 3 | 2 |
| Gregoire et al. | 2022 | 107 | 0.4160 | 26.00 | 76.60 | 1 | 87% Canadian | 5 | 49.00 | 7 | 12 | 3 | 2 |
| Suffoleto et al. | 2021 | 52 | 0.0730 | 18.70 | 79.00 | 1 | 97% White, 3% Black, 6% Hispanics | 6 | 91.20 | 12 | 12 | 1 | 3 |
| Suffoleto et al. | 2021 | 52 | 0.2140 | 18.70 | 79.00 | 1 | 97% White, 3% Black, 6% Hispanics | 6 | 91.20 | 12 | 12 | 3 | 3 |
| Sin et al. | 2022 | 407 | 0.1370 | 53.10 | 81.00 | 1 | 87% White, 4% Mixed, 5% Asian, 4% Black, 1% other | 4 | 85.00 | 40 | 40 | 3 | 4 |
| Baumel et al. | 2018 | 19 | 0.9750 | 31.95 | - | 1 | 40% African American, 25% White, 15% Hispanic, 10% Asian, 10% multiracial | 4 | 85.00 | 4 | - | 1 | 2 |
| Birrell et al. | 2023 | 166 | 0.0590 | 15.20 | 43.30 | 2 | 90.1% born in Australia | 1 | 77.30 | 48 | 48 | 1 | 2 |
| Birrell et al. | 2023 | 166 | 0.0930 | 15.20 | 43.30 | 2 | 90.1% born in Australia | 1 | 77.30 | 48 | 48 | 3 | 2 |
| Fortuna et al. | 2022 | 21 | 0.5430 | 37.30 | 66.60 | 1 | 21% White | 1 | 70.00 | 48 | - | 1 | 2 |
| Klimczak et al. | 2023 | 230 | 0.4530 | 21.70 | 76.00 | 1 | 89.3% White, 1.3% Hispanic, 2.7% Asian, 1.3% Native Hawaiian/Pacific Islander | 6 | 74.00 | 40 | - | 1 | 2 |
| Klimczak et al. | 2023 | 230 | 0.5520 | 21.70 | 76.00 | 1 | 89.3% White, 1.3% Hispanic, 2.7% Asian, 1.3% Native Hawaiian/Pacific Islander | 6 | 74.00 | 40 | - | 3 | 2 |
| Klimczak et al. | 2023 | 230 | 0.0090 | 21.70 | 76.00 | 1 | 89.3% White, 1.3% Hispanic, 2.7% Asian, 1.3% Native Hawaiian/Pacific Islander | 6 | 74.00 | 40 | - | 3 | 2 |
| Wright et al. | 2022 | 40 | 0.8000 | 48.80 | 83.00 | 1 | 91.3% White British, 6.5% Asian British, 2.2% prefer not to say | 5 | 70.00 | 32 | - | 1 | 1 |
| Drysdale et al. | 2021 | 90 | 0.9520 | 17-23 | 36.70 | 1 | - | 5 | 68.60 | 24 | - | 1 | 3 |
| Drysdale et al. | 2021 | 90 | 0.3380 | 17-26 | 39.70 | 1 | - | 5 | 68.60 | 24 | - | 3 | 3 |
| Drysdale et al. | 2021 | 90 | 0.9340 | 17-27 | 40.70 | 1 | - | 5 | 68.60 | 24 | - | 3 | 3 |
| Pauksztat et al. | 2022 | 131 | 0.9780 | 20.32 | 67.90 | 1 | 34.1% from the Philippines, followed by Denmark, Germany and Sweden (7.4% each) | 4 | 100.00 | 8 | 8 | 3 | 1 |
| Tomasino et al. | 2017 | 47 | 0.9920 | 69.60 | 68.10 | 1 | 4.3% African American, 87.2% White, 6.4% more than 1 race, 2.1% declined to report | 4 | 83.00 | 2 | - | 1 | 3 |
| Tomasino et al. | 2017 | 47 | 0.3600 | 69.60 | 68.10 | 1 | 4.3% African American, 87.2% White, 6.4% more than 1 race, 2.1% declined to report | 4 | 83.00 | 2 | - | 3 | 3 |
| Tomasino et al. | 2017 | 47 | 0.3640 | 69.60 | 68.10 | 1 | 4.3% African American, 87.2% White, 6.4% more than 1 race, 2.1% declined to report | 4 | 83.00 | 2 | - | 3 | 3 |
| Schulz et al. | 2016 | 148 | 0.7480 | 35.38 | 53.00 | 1 | - | 4 | 53.00 | 12 | 24 | 1 | 4 |
| Schulz et al. | 2016 | 148 | 0.3880 | 35.38 | 53.00 | 1 | - | 4 | 53.00 | 12 | 24 | 3 | 4 |
| Schulz et al. | 2016 | 148 | 0.0080 | 35.38 | 53.00 | 1 | - | 4 | 53.00 | 12 | 24 | 3 | 4 |
| Hensel et al. | 2019 | 1455 | 0.4900 | 41.40 | 73.00 | 1 | 82% White, 17% non-white | 4 | 48.00 | 12 | - | 1 | 1 |
| Hensel et al. | 2019 | 1455 | 0.4090 | 41.40 | 73.00 | 1 | 82% White, 17% non-white | 4 | 48.00 | 12 | - | 3 | 1 |
| Nosek et al. | 2016 | 19 | 0.7400 | 43.21 | 100.00 | 1 | 52.6% Caucasian, 21.1% Hispanics, 21.1% African American, 5.3% others | 2 |  | 1 | - | 1 | 1 |
| Setoyama et al. | 2011 | 465 | 0.1020 | 43.71 |  | 2 | - | 4 | 54.00 | 4 | - | 3 | 1 |
| Lepore et al. | 2019 | 183 | 0.9180 | 29-65 | 100.00 | 1 | 95.6% White | 2 | 87.00 | 4 | - | 1 | 1 |
| Pretorius et al. | 2009 | 101 | 0.932 | 18.80 | 97.00 | 1 | - | 2 | 70.00 | 12 | 24 | 1 | 4 |
| McKay et al. | 2001 | 78 | 0.166 | 52.30 | 82.00 | 1 | - | 4 | 87.00 | 8 | - | 1 | 3 |
| McKay et al. | 2001 | 78 | 0.374 | 52.30 | 82.00 | 1 | - | 4 | 87.00 | 8 | - | 3 | 3 |
| Freeman et al. | 2008 | 238 | 0.408 | 21.00 | 70.00 | 1 | The largest ethnic group was White (71%), and the next largest was UK Asian (i.e., from the Indian subcontinent: 71, 10%). | 4 | - | 10 | - | 1 | 1 |
| Freeman et al | 2008 | 238 | 0.232 | 21.00 | 70.00 | 1 | The largest ethnic group was White (71%), and the next largest was UK Asian (i.e., from the Indian subcontinent: 71, 10%). | 4 | - | 10 | - | 3 | 1 |
| Wikerson et al | 2018 | 12 | 0.620 | 55,7 | 58.30 | 1 | 75% Caucasian, 25% African American | 3 | 38.30 | 6 | 24 | 1 | 1 |
| Houston et al. | 2002 | 103 | 0.548 | 18-over 45 | 81.00 | 1 | 18 participants were from Canada, Australia, or Europe | 2 | 69.00 | 2 | - | 3 | 1 |
| Mouthan et al. | 2013 | 300 | 0.027 | 44.18 | 41.10 | 1 | 84.1% Dutch cultural background | 2 | 46.00 | 4 | 48 | 1 | 2 |
| Mouthan et al. | 2013 | 300 | 0.149 | 44.18 | 41.10 | 1 | 84.1% Dutch cultural background | 2 | 46.00 | 4 | 48 | 3 | 2 |
| Nelson et al. | 2014 | 19 | 0.299 | 50.47 | 0.00 | 1 | 68.4% Caucasians | 4 | 74.00 | 4 | 8 | 1 | 2 |
| Stevens et al. | 2022 | 302 | 0.475 | 16.70 | 79.10 | 1 | 82.5% White, 7.1% Asian, 6.4% Mixed, 4% Black | 4 | 48.00 | 4 | - | 1 | 4 |
| Stevens et al. | 2022 | 302 | 0.082 | 16.70 | 79.10 | 1 | 82.5% White, 7.1% Asian, 6.4% Mixed, 4% Black | 4 | 48.00 | 4 | - | 4 | 4 |
| Yeung et al. | 2021 | 253 | 0.209 | 37.50 | 95.20 | 2 | 100% Chinese | 5 | 72.00 | 8 | - | 1 | 3 |
| Houwen et al. | 2010 | 253 | 0.162 | 42.97 | 93.70 | 1 | - | 5 | 100.00 | 12 | 24 | 3 | 1 |
| Houwen et al. | 2010 | 253 | 0.156 | 42.97 | 93.70 | 1 | - | 2 | 100.00 | 12 | 24 | 3 | 1 |
| Bautista et al. | 2022 | 35 | 0.622 | 21.86 | 71.40 | 1 | 71.4%, European American, 11.4%, African American, 8.6% Hispanic 17.1% Asian American | 4 | 80.00 | 6 | - | 3 | 2 |
| Bautista et al. | 2022 | 35 | 0.426 | 21.86 | 71.40 | 1 | European American 71.4%, African American 11.4%, Hispanic 8.6%, Asian American 17.1% | 4 | 90.00 | 6 | - | 3 | 2 |
| Joyce et al. | 2018 | 29 | 0.288 | 43.70 | 3.00 | 2 | - | 4 | 38.00 | 1 | - | 1 | 2 |
| Linke | 2007 | 10000 | 0.778 | 37.40 | 51.10 | 1 | 81.9% White British, 9.1% White other, 5.3% White Irish, 1.4% Asian, 0.9% Mixed, 0.7% Black, 0.8% Other | 2 | 17.00 | 6 | - | 1 | 1 |
| Paulson & Casile | 2015 | 6 | 0.340 | 29-83 |  | 1 | - | 2 | 100.00 | 24 | - | 1 | 2 |
| Kramer et al. | 2015 | 270 | 0.187 | 42.90 | 87.20 | 1 | - | 4 | 87.00 | 48 | 48 | 1 | 1 |
| Ebert et al. | 2013 | 400 | 0.968 | 45.09 | 73.50 | 1 | - | 4 | 65.50 | 12 | 48 | 1 | 4 |
| Ebert et al. | 2013 | 400 | 0.000 | 45.09 | 73.50 | 1 | - | 4 | 65.50 | 12 | 48 | 3 | 4 |
| Owen et al. | 2016 | 299 | 0.005 | 53.80 | 77.70 | 1 | - | 2 |  | 12 | - | 1 | 1 |
| Niemiec et al. | 2018 | 37 | 0.400 | 58.20 | 0.91 | 3 | 2.7% US, 64.9% Mexico, 32.4% others | 1 | 92.50 | 16 | - | 1 | 2 |
| Niemiec et al. | 2018 | 37 | 0.545 | 58.20 | 0.91 | 3 | 2.7% US, 64.9% Mexico, 32.4% others | 1 | 92.50 | 16 | - | 1 | 2 |
| Goldberg et al. | 2015 | 2817 | 0.181 | 42.00 |  | 1 | - | 4 | 84.00 | 48 | - | 1 | 1 |
| Goldberg et al. | 2015 | 2817 | 0.039 | 42.00 |  | 1 | - | 4 | 84.00 | 96 | - | 1 | 1 |
| Imanaka et al. | 2013 | 193 | 0.698 | 50.00 | 13.40 | 2 | - | 4 | 89.70 | 12 | - | 4 | 4 |
| Zheng et al. | 2021 | 954 | 0.089 | 13.50 | 48.90 | 2 | - | 1 | 63.00 | 2 | - | 3 | 1 |
| Zheng et al. | 2021 | 954 | 0.112 | 13.50 | 48.90 | 2 | - | 1 | 63.00 | 2 | - | 3 | 1 |
| Kelly et al. | 2021 | 72 | 0.410 | 44.00 | 39.00 | 2 | 81% born in Australia Aboriginal, 6% Torres Strait, or both Aboriginal and Torres Strait Islander descent | 1 | 100.00 | 8 | - | 1 | 4 |
| Morriss et al. | 2021 | 790 | 0.220 | 37.60 | 81.00 | 1 | 93.4% White, 2% South Asian, 0.7% Black, 3.6% others | 4 | 92.00 | 24 | - | 4 | 1 |
| Smit et al. | 2022 | 301 | 0.440 | 50.20 | 66.10 | 1 | - | 2 | 24.60 | 12 | 24 | 1 | 1 |
| Shorey et al. | 2019 | 138 | 0.960 | 32.05 | 100.00 | 2 | - | 1 | 79.70 | 4 | 12 | 3 | 2 |
| Shorey et al. | 2019 | 138 | 0.824 | 32.05 | 100.00 | 2 | - | 1 | 79.70 | 12 | 12 | 3 | 2 |
| Bravata et al. | 2023 | 815 | 0.569 | 38.00 | 3.81 | 1 | 53.7% White, 16.3% Hispanics, 6.3% Black | 4 | 73.00 | 12 | - | 1 | 4 |
| Houston et al. | 2002 | 103 | 0.587 | 40.00 | 78.60 | 1 | - | 2 | 100.00 | 24 | 48 | 1 | 1 |
| Houston et al. | 2002 | 103 | 0.505 | 40.00 | 78.60 | 1 | - | 2 | 100.00 | 48 | 48 | 4 | 1 |
| Dennis et al. | 2009 | 701 | 0.129 | 20-35 | 100.00 | 1 | - | 6 | 82.80 | 12 | 24 | 3 | 2 |
| Dennis et al. | 2009 | 701 | 0.067 | 20-35 | 100.00 | 1 | - | 6 | 82.80 | 12 | 24 | 3 | 2 |
| Dennis et al | 2009 | 701 | 0.133 | 20-36 | 100.00 | 1 | - | 6 | 82.80 | 24 | 24 | 4 |  |
| Linke et al. | 2007 | 8933 | 0.597 | 37.40 | 0.51 | 3 | (83.9%) lived in the United Kingdom, and 9.3% reported living in other English-speaking countries (United States, Canada, Australia, and New Zealand). Over 100 countries of residence were given by the remaining 6.7% of users. 81.9%, White British, 9.1% White other, 5.3% White Irish, 0.9% Asian, 0.7% Mixed 1.4% Black,  0.8% other | 2 | 18.50 | 6 | - | 1 | 1 |
| Baustita et al. | 2022 | 35 | 0.082 | 21.86 | 71.40 | 1 | - | 4 | 100.00 | 6 | - | 3 | 2 |
| Baustita et al. | 2022 | 35 | 0.763 | 21.86 | 71.40 | 1 | - | 4 | 100.00 | 6 | - | 3 | 2 |
| Stevens et al. | 2022 | 302 | 0.410 | 16.70 | 79.10 | 1 | 82.5% White, 7.1% Asian, 6.4% mixed, 4% Black | 4 | 100.00 | 4 | - | 4 | 4 |
| Yeung et al. |  | 253 | 0.490 | 37.80 | 97.80 | 2 | - | 5 | 83.30 | 8 | - | 1 | 2 |
| Houwen et al. | 2010 | 253 | 0.164 | 42.97 | 93.70 | 1 | - | 2 | 87.00 | 1 | - | 3 | 1 |
| Kruzan et al. | 2022 | 131 | 0.479 | 20.32 | 67.90 | 1 | 62.6% North America, 18.3% European Union, 19.1% UK | 1 | 92.00 | 8 | 16 | 3 | 1 |
| Kruzan et al. | 2022 | 131 | 0.163 | 20.32 | 67.90 | 1 | 62.6% North America, 18.3% European Union, 19.1% UK | 1 | 92.00 | 16 | 16 | 3 | 1 |
| Gillard et al. | 2022 | 590 | 0.040 | 39.70 | 0.52 | 1 | 13% Asian British, 16% Black British, 11% mixed & other, 58% White | 6 | 87.60 | 16 | 48 | 3 | 3 |
| Gillard et al. | 2022 | 590 | 0.028 | 39.70 | 0.52 | 1 | 13% Asian British, 16% Black British, 11% mixed & other, 58% White | 6 | 97.60 | 48 | 48 | 3 | 3 |
| Klimczak et al. |  | 230 | 0.453 | 22.00 | 75.00 | 1 | 87% White, 2.6% Hispanic, 2.6% African American, 1.3% Pacific Islander 5.2% Multiracial, 1.3% prefer not to share | 6 | 76.00 | 10 | - | 1 | 2 |
| Klimczak et al. |  | 230 | 0.591 | 22.00 | 75.00 | 1 | 87% White, 2.6% Hispanic, 2.6% African American, 1.3% Pacific Islander 5.2% Multiracial, 1.3% prefer not to share | 6 | 76.00 | 10 | - | 3 | 2 |
| Klimczak et al. |  | 230 | 0.009 | 22.00 | 75.00 | 1 | 87% White, 2.6% Hispanic, 2.6% African American, 1.3% Pacific Islander 5.2% Multiracial, 1.3% prefer not to share | 6 | 76.00 | 10 | - | 4 | 2 |
| Birrel et al. | 2023 | 166 | 0.102 | 15.30 | 43.40 | 3 | 86% Australia, 14% other country | 1 | 77.30 | 48 | - | 3 | 1 |
| Birrel et al. | 2023 | 166 | 0.278 | 15.30 | 43.40 | 3 | 86% Australia, 14% other country | 1 | 77.30 | 48 | - | 3 | 1 |
| Birrel et al. | 2023 | 166 | 0.238 | 15.30 | 43.40 | 3 | 86% Australia, 14% other country | 1 | 77.30 | 48 | - | 3 | 1 |
| Summers et al. | 2021 | 347 | 0.577 | 49.60 | 59.30 | 1 | - | 1 | 79.00 | 12 | - | 1 | 1 |
| Kahl et al. | 2020 | 1982 | 0.325 | 19.40 | 83.60 | 1 | - | 4 | 81.00 | 12 | - | 1 | 4 |
| Hensel et al. | 2019 | 542 | 0.140 | 42.20 |  | 1 | 82.4% White, 17.6% non-White | 4 | 43.00 | 12 | - | 4 | 1 |
| Economides et al. | 2019 | 102 | 0.390 | 32.90 | 77.50 | 1 | 78.4%, Finland, 21.6% US | 1 | 100.00 | 8 | 48 | 1 | 3 |
| Economides et al. | 2019 | 102 | 0.495 | 32.90 | 77.50 | 1 | 78.4%, Finland, 21.6% US | 1 | 100.00 | 48 | - | 1 | 3 |
| Moir et al. | 2016 | 275 | 0.122 | 21.00 | 0.53 | 1 | - | 6 | 85.20 | 24 | - | 3 | 2 |
| Moir et al. | 2016 | 275 | 0.038 | 21.00 | 0.53 | 1 | - | 6 | 85.20 | 24 | - | 3 | 2 |
| Moir et al. | 2016 | 275 | 0.065 | 21.00 | 0.53 | 1 | - | 6 | 85.20 | 24 | - | 3 | 2 |

*Note*. SMD=Standardized mean difference; N = sample size; Gender = percentage of sample who are female; Country = sample’s cultural background (1 = Western; 2 = Eastern; 3 = A combination different cultural background); Platform = mode of peer support (1 = mobile apps; 2 = forums and discussion boards; 3 = social networking sites; 4 = websites; 5 = videoconference; 6 = email, texts messages, and phone calls); Duration = duration of intervention (in weeks); Follow-up = follow-up measurements since the first measurement (in weeks); Study design (1 = pre-post digital peer support comparison; 3 = digital peer support vs. control; 4 = digital peer support vs. alternative digital intervention); Source of digital peer support (1 = Informal naturally occurring; 2 = Formal unpaid and/or paid; 3 = Professional support; 4 = A mix of peer and professional support). Overall, these 73 studies randomized 52,703 participants to these conditions: informal naturally occurring peer support (n=28/73; 38.4%), formal unpaid/paid peer support (n=21/73; 28.8%), professional support (n=14/73; 19.2%), a mix of peer and professional support (n=9/73; 12.3%)^[[1]](#footnote-1)^. The 73 studies originated from 14 countries, for instance, Australia, Canada, Germany, Italy, Singapore, Sweden, Taiwan, UK, US, and China. Most of the study participants were from the United States of America or countries in Europe and Australia (n = 63/73; 86.3%). The mean age of the sample for the examined studies ranged from 13.50 to 69, and the female proportion ranged from 0 to 100. The mean duration of intervention was 16.29 weeks (SD=15.90; range=1 to 96 weeks). 41 (56.2%) interventions examined pre-post digital peer support comparisons, 34 (46.6%) examined digital peer support vs. control, and 12 (16.4%) examined digital peer support vs. alternative digital interventions. Fifty-nine studies (80.8%) measured post-intervention effects without follow-up, and 14 (19.2%) studies included both post-intervention and long-term effects. The mean follow-up assessment was 26.4 weeks and the range was 3 weeks to 48 weeks.

Table A6

Characteristics of Digital Peer Support Interventions and Physical Health

| **Authors** | **Year** | **N** | **SMD** | **Mean**  **age** | **Gender** | **Country** | **Ethnic Composition** | **Plat form** | **Uptake (100%)** | **Duration** | **Follow up assessment** | **Study Design** | **Source of support** |
| --- | --- | --- | --- | --- | --- | --- | --- | --- | --- | --- | --- | --- | --- |
| DeBar et al. | 2009 | 228 | 0.131 | 15.60 | 100.00 | 1 | 80.5% White | 2 | 100.00 | 96 | -- | 3 | 4 |
| Goldberg et al. | 2015 | 2817 | 0.219 | 42.00 |  | 1 | -- | 4 | 84.00 | 48 | 96 | 1 | 1 |
| Goldberg et al. | 2015 | 2817 | 0.250 | 42.00 |  | 1 | -- | 4 | 84.00 | 96 | -- | 1 | 1 |
| Mamede et al. | 2021 | 298 | 0.060 | 47.50 | 55.30 | 1 | 89.4% Dutch | 1 | 79.00 | 10 | 14 | 3 | 1 |
| Mamede et al. | 2021 | 298 | 0.043 | 47.50 | 55.30 | 1 | 89.4% Dutch | 1 | 79.00 | 14 | -- | 3 | 1 |
| Keyserling et al. | 2008 | 236 | 0.346 | 54.00 | 100.00 | 1 | 58% White, 41% African American | 6 | 65.00 | 24 | 48 | 4 | 2 |
| Keyseling et al. | 2008 | 236 | 0.555 | 54.00 | 100.00 | 1 | 58% White, 41% African American | 6 | 65.00 | 48 | -- | 4 | 2 |
| Lara et al. | 2016 | 75 | 0.079 | 60.90 | 76.00 | 1 | -- | 4 | 98.00 | 8 | -- | 3 | 1 |
| Lara et al. | 2016 | 75 | 0.238 | 60.90 | 76.00 | 1 | -- | 4 | 98.00 | 8 | -- | 3 | 1 |
| Moravcova et al. | 2022 | 100 | 0.333 | 43.00 | 0.71 | 1 | -- | 1 | 49.00 | 48 | 24 | 3 | 1 |
| Moravcova et al. | 2022 | 100 | 0.062 | 43.00 | 0.71 | 1 | -- | 1 | 49.00 | 24 | -- | 3 | 1 |
| Moravcova et al. | 2022 | 100 | 0.179 | 43.00 | 0.71 | 1 | -- | 1 | 49.00 | 48 | -- | 3 | 1 |
| Tsai & Liu | 2015 | 105 | 0.236 | 35.80 | 100.00 | 2 | -- | 2 | 91.30 | 12 | -- | 3 | 4 |
| Tsai & Liu | 2015 | 105 | 0.312 | 35.80 | 100.00 | 2 | -- | 2 | 91.30 | 12 | -- | 3 | 4 |
| Mi et al. | 2022 | 268 | 0.163 | 42.33 | 47.00 | 1 | 66% White, 24% African American, 6% others, 5% American Indian, 0.004% Asian | 1 | 78.00 | 24 | -- | 1 | 1 |
| Sharps et al. | 2019 | 20 | 0.417 | 19.00 | 0.95 | 1 | -- | 3 | 54.90 | 2 | -- | 3 | 2 |
| Sharps et al. | 2019 | 20 | 0.382 | 19.00 | 0.95 | 1 | -- | 3 | 54.90 | 2 | -- | 3 | 2 |
| West et al. | 2016 | 58 | 0.544 | 21.60 | 81.00 | 1 | -- | 3 | 90.00 | 9 | -- | 3 | 1 |
| West et al. | 2016 | 58 | 0.834 | 21.60 | 81.00 | 1 | -- | 3 | 90.00 | 9 | -- | 3 | 1 |
| Watanabe-Ito et al | 2020 | 42 | 0.364 | 20.10 | 100.00 | 2 | -- | 1 | 90.50 | 1 | 4 | 1 | 1 |
| Watanabe-Ito et al | 2020 | 42 | 0.202 | 20.10 | 100.00 | 2 | -- | 1 | 90.50 | 4 | 4 | 1 | 1 |
| Carlsen et al. | 2013 | 226 | 0.446 | 31.30 | 100.00 | 1 | -- | 6 | 97.20 | 24 | -- | 3 | 3 |
| Carlsen et al. | 2013 | 226 | 0.057 | 31.30 | 100.00 | 1 | -- | 6 | 97.20 | 24 | -- | 3 | 3 |
| Morgan et al. | 2011 | 65 | 0.051 | 35.90 | 0.00 | 1 | -- | 4 | 76.50 | 12 | 48 | 3 | 1 |
| Morgan et al. | 2011 | 65 | 0.159 | 35.90 | 0.00 | 1 | -- | 4 | 76.50 | 48 | -- | 3 |  |
| Leahey et al. | 2016 | 75 | 0.164 | 48.50 | 85.30 | 1 | 1.3% Hispanic, 97.3% not Hispanic | 4 | 100.00 | 40 | -- | 4 | 4 |
| Leahey et al. | 2016 | 75 | 0.730 | 48.50 | 85.30 | 1 | 1.3% Hispanic, 97.3% not Hispanic | 4 | 100.00 | 40 | -- | 3 | 4 |
| Tate et al. | 2006 | 192 | 0.825 | 49.20 | 0.84 | 1 | -- | 2 | 81.30 | 12 | 24 | 1 | 1 |
| Tate et al. | 2006 | 192 | 0.031 | 49.20 | 0.84 | 1 | -- | 2 | 81.30 | 24 | -- | 1 | 1 |
| Tate et al. | 2006 | 192 | 0.193 | 49.20 | 0.84 | 1 | -- | 2 | 81.30 | 12 | -- | 4 | 1 |
| Tate et al. | 2006 | 192 | 0.136 | 49.20 | 0.84 | 1 | -- | 2 | 81.30 | 12 | -- | 3 | 1 |
| Harvey-Berino et al. | 2004 | 255 | 0.170 | 45.80 | 0.82 | 1 | -- | 6 | 67.50 | 48 | -- | 4 | 4 |
| Lim et al. | 2020 | 108 | 0.373 | 46.80 | 42.00 | 2 | 70.4% Chinese 10.2% Malay, 6.5% Indian, 5.6% others | 4 | 90.00 | 24 | -- | 3 | 1 |
| Ross et al. | 2022 | 3623 | 0.602 | 57.80 | 50.00 | 1 | 16% Asian, 5% Black, 3% mixed, 0.4% others, 68% White | 1 |  | 48 | -- | 4 | 1 |
| Fiks et al. | 2017 | 87 | 0.450 | 26.50 | 100.00 | 1 | 5% Hispanic, 84% African American, 7% White, 7% Others | 3 | 88.40 | 24 | -- | 1 | 1 |
| Tate et al. | 2001 | 91 | 0.400 |  | 0.89 | 1 | -- | 6 | 78.00 | 24 | -- | 3 | 3 |
| Pretlow et al. | 2015 | 43 | 0.700 | 16.00 | 0.65 | 1 | 84% Caucasian, 9.3% African American, 4.7% Latino 2.3% Asian | 1 | 62.80 | 20 | -- | 1 | 1 |
| Webber et al. | 2008 | 66 | 0.055 | 50.00 | 100.00 | 1 | 86% Caucasian | 4 | 100.00 | 16 | -- | 4 | 4 |
| Webber et al. | 2008 | 66 | 0.102 | 50.00 | 100.00 | 1 | 86% Caucasian | 4 | 100.00 | 16 | -- | 4 | 4 |
| Pappa et al. | 2017 | 107886 | 0.830 |  | 92.90 | 3 | -- | 3 | 100.00 | 4 | -- | 1 | 1 |
| Anderson | 2022 | 80 | 0.110 | 22.20 | 80.00 | 1 | 66.3% White, 17.5 % Black, 6.3% Asian, 6.3% Biracial, 3.8% Latino | 3 | 89.00 | 12 | -- | 3 | 1 |
| Anderson | 2022 | 80 | 0.117 | 22.20 | 80.00 | 1 | 66.3% White, 17.5 % Black, 6.3% Asian, 6.3% Biracial, 3.8% Latino | 3 | 89.00 | 12 | -- | 3 | 2 |
| Anderson | 2022 | 80 | 0.049 | 22.20 | 80.00 | 1 | 66.3% White, 17.5 % Black, 6.3% Asian, 6.3% Biracial, 3.8% Latino | 3 | 89.00 | 12 | -- | 4 | 3 |
| Johnson & Wardle | 2011 | 3621 | 0.043 | 35.50 | 82.00 | 1 | -- | 4 | 100.00 | 20 | -- | 3 | 1 |
| Turner-McGrievy et al. | 2013 | 47 | 0.480 | 42.60 | 77.00 | 1 | 25% non-White, 75% White | 3 | 100.00 | 24 | -- | 4 | 4 |
| Lee et al. | 2018 | 22 | 0.384 | 50.90 | 100.00 | 1 | -- | 6 | 77.00 | 20 | -- | 1 | 2 |
| Dennison et al. | 2014 | 786 | 0.086 | 44.00 | 79.70 | 1 | -- | 4 | 100.00 | 8 | -- | 3 | 1 |
| Dennison et al. | 2014 | 786 | 0.020 | 44.00 | 79.70 | 1 | -- | 4 | 100.00 | 8 | -- | 4 | 2 |
| Hageman et al. | 2017 | 301 | 0.092 | 53.90 | 100.00 | 1 | 98% White, 1% Hispanic, 1% other | 2 | 67.00 | 120 | -- | 3 | 1 |
| Hageman et al. | 2017 | 301 | 0.069 | 53.90 | 100.00 | 1 | 98% White, 1% Hispanic, 1% other | 2 | 67.00 | 121 | -- | 4 | 1 |
| Richardson et al. | 2010 | 324 | 0.388 | 52.00 | 65.00 | 1 | 86% White, 6% Black, 3% American Indian, 1% others | 2 | 79.10 | 16 | -- | 3 | 1 |
| An et al. | 2013 | 1698 | 0.051 | 24.07 | 72.00 | 1 | 73.91% White, 10.36% African American, 8.6% other, 7.13% multiple | 2 | 76.10 | 6 | 12 | 1 | 2 |
| Rosas et al. | 2022 | 200 | 0.438 | 47.30 | 0.00 | 1 | -- | 5 | 100.00 | 12 | 32 | 1 | 1 |
| Li et al. | 2021 | 353 | 0.386 |  | 57.79 | 2 | -- | 4 | 100.00 |  | -- | 1 | 1 |
| Imanaka et al. | 2013 | 193 | 0.282 | 50.00 | 13.40 | 2 | -- | 4 | 89.70 | 12 | -- | 4 | 4 |
| Sepah et al. | 2017 | 220 | 0.691 | 43.60 | 82.70 | 1 | 49.1% White, 28.6% Black, 10.5% Hispanic, 9.5% others, 2.3% undisclosed | 3 | 46.40 | 12 | 144 | 1 | 4 |
| Sepah et al. | 2017 | 220 | 0.290 | 43.60 | 82.70 | 1 | 49.1% White, 28.6% Black, 10.5% Hispanic, 9.5% others, 2.3% undisclosed | 3 | 46.40 | 144 | -- | 1 | 4 |
| Ali et al. | 2021 | 79 | 0.887 | 61.50 |  | 1 | -- | 5 | 84.00 | 10 | -- | 1 | 4 |
| Ali et al. | 2021 | 79 | 0.536 | 62.50 |  | 2 | -- | 5 | 84.00 | 10 | -- | 1 | 4 |
| Travis et al. | 2010 | 54 | 0.375 | 52.40 | 37.00 | 1 | -- | 5 | 59.30 | 12 | -- | 1 | 1 |
| Travis et al. | 2010 | 54 | 0.258 | 52.40 | 37.00 | 1 | -- | 5 | 59.30 | 12 | -- | 1 | 1 |
| Hensel et al. | 2019 | 812 | 0.182 | 41.50 | 73.00 | 1 | 82% White, 17% non-White | 4 | 46.00 | 12 | -- | 3 | 1 |
| Hensel et al. | 2019 | 812 | 0.125 | 41.50 | 73.00 | 1 | 82% White, 17% non-White | 4 | 46.00 | 12 | -- | 3 | 1 |
| Dennis et al. | 2009 | 701 | 0.165 |  | 100.00 | 1 | -- | 6 | 82.80 | 12 | 24 | 3 | 2 |
| Dennis et al. | 2009 | 701 | 0.019 |  | 100.00 | 1 | -- | 6 | 82.80 | 12 | 24 | 3 | 2 |
| Dennis et al. | 2009 | 701 | 0.562 |  | 100.00 | 1 | -- | 6 | 82.80 | 24 | 24 | 4 |  |
| Linke et al. | 2007 | 8933 | 0.634 | 37.40 | 0.51 | 3 | (83.9%) lived in the United Kingdom, and 9.3% reported living in other English-speaking countries (United States, Canada, Australia, and New Zealand). Over 100 countries of residence were given by the remaining 6.7% of users. 81.9% White British, 9.1% White other, 5.3% White Irish, 1.4% Asian, 0.9% Mixed, 0.7% Black, 0.8% other | 2 | 18.50 | 6 | -- | 1 | 1 |
| Oween et al. | 2016 | 299 | 0.496 | 53.80 | 77.70 | 1 | -- | 3 | 100.00 | 12 | -- | 3 | 1 |
| Stevens et al. | 2022 | 302 | 0.769 | 16.70 | 79.10 | 1 | 82.5% White, 7.1% Asian, 6.4% mixed, 4% Black | 4 | 100.00 | 4 | -- | 4 | 4 |
| Paulson et al. | 2015 | 6 | 0.341 |  |  | 1 | -- | 2 | 100.00 | 24 | -- | 1 | 4 |
| Freeman et al. | 2008 | 238 | 0.408 | 21.00 | 70.00 | 1 | US, the largest ethnic group was White (71%), and the next largest was UK Asian (i.e., from the Indian subcontinent: 10%) | 2 | 100.00 | 10 | -- | 4 | 1 |
| Freeman et al. | 2008 | 238 | 0.232 | 21.00 | 70.00 | 1 | US, the largest ethnic group was White (71%), and the next largest was UK Asian (i.e., from the Indian subcontinent: 10%) | 2 | 100.00 | 10 | -- | 4 | 1 |
| Mckeon et al. | 2021 | 24 | 0.496 | 48.10 | 0.17 | 1 | -- | 3 | 88.00 | 10 | -- | 1 | 3 |
| Gould et al. | 2021 | 54 | 0.177 | 57.06 | 0.60 | 1 | 6% Hispanic , 16% Asian, 10% Black, 58% White, 8% other | 1 | 83.33 | 12 | -- | 1 | 4 |
| Suffoleto et al. | 2021 | 52 | 0.238 | 18.70 | 79.00 | 1 | 97% White, 3% Black, 6% Hispanics | 6 | 91.2 | 12 | 12 | 3 | 3 |
| Suffoleto et al. | 2021 | 52 | 0.355 | 18.70 | 79.00 | 1 | 97% White, 3% Black, 6% Hispanics | 6 | 91.2 | 12 | 12 | 3 | 3 |

*Note*. SMD=Standardized mean difference; N = sample size; Gender = percentage of sample who are female; Country = sample’s cultural background (1 = Western; 2 = Eastern; 3 = A combination different cultural background); Platform = mode of peer support (1 = mobile apps; 2 = forums and discussion boards; 3 = social networking sites; 4 = websites; 5 = videoconference; 6 = email, texts messages, and phone calls); Duration = duration of intervention (in weeks); Follow-up = follow-up measurements since the first measurement (in weeks); Study design (1 = pre-post digital peer support comparison; 3 = digital peer support vs. control; 4 = digital peer support vs. alternative digital intervention); Source of digital peer support (1 = Informal naturally occurring; 2 = Formal unpaid and/or paid; 3 = Professional support; 4 = A mix of peer and professional support). Overall, these 47 studies randomized 145,326 participants to these conditions: informal naturally occurring peer support (n=26/47; 55.3 %), formal unpaid/paid peer support (n=7/46; 15.2%), professional support (n=5/47; 10.6%), a mix of peer and professional support (n=12/47; 25.5%)^[[2]](#footnote-2)^. The 47 studies originated from 14 countries, for instance, Australia, Canada, Britain, Czech Republic, Netherlands, Singapore, Japan, Taiwan, UK, US, and China. Most of the study participants were from the United States of America or countries in Europe and Australia (n = 38/47; 80.9%). The mean age of the sample for the examined studies ranged from 15.60 to 62.5, and the female proportion ranged from 0 to 100. The mean duration of intervention was 24.08 weeks (SD=27.86; range=1 to 144 weeks). 18 (38.3%) interventions examined pre-post digital peer support comparisons, 20 (42.6%) examined digital peer support vs. control, and 14 (29.8%) examined digital peer support vs. alternative digital interventions. Thirty-seven studies (78.7%) measured post-intervention effects without follow-up, and 10 (21.3%) studies included both post-intervention and long-term effects. The mean follow-up assessment was 34.1 weeks and the range was 4 weeks to 144 weeks.

Multiple effect sizes

First, for both physical and mental health, cases examining more than one source of digital support, the different effect sizes were used to conduct analyses separately: (a) informal naturally occurring support, (b) formal unpaid and/or paid support peer support, and (c) professional support. Second, some studies included multiple indicators of physical and mental health. Thus, there were several effect sizes from the same study. For such cases, we computed the average effect size across all measures of the same physical and mental health outcome within a study. Hence, each study contributed only one effect size for the analyses involving each source of digital peer support with physical and mental health. Third, a few studies reported multiple effect sizes because they investigated different countries, especially concerning representatives of Western and Eastern cultural contexts. Because cultural contexts were examined as moderators in our meta-analyses, a single effect size estimate that aggregated the multiple correlation coefficients was not favoured and we reported effect size estimates separately (Hunter & Schmidt, 2014). For such cases, more than one set of data was collected from the same study, forcing consideration of issues of statistical dependency that stem from the multiple dependent effect sizes (Hunter & Schmidt, 2004). We used the robust variance estimation to account for non-independent effect sizes, which can also be adjusted to deal with smaller meta-analyses (n < 40; Tipton, 2015).

Risk of bias (quality) assessment

Studies were independently evaluated for quality by two reviewers with differences discussed and resolved using the Cochrane Collaboration’s risk of bias tool, with seven domains of bias: random sequence generation, allocation concealment, blinding of participants and personnel, blinding of outcome assessment, incomplete outcome data, selective reporting, and other bias. Of the 149 interventions, only 17 (11.4%) had low risk of bias ratings across the seven categories. 136 studies (91.3%) used random sequence generation, 95 (63.8%) had allocation concealment, blinding of participants and personnel, blinding of outcome assessment, incomplete outcome data, selective reporting, and other bias. Only 9 (6%) blinded both participants and personnel to the condition allocation (performance bias), 57 (38.3%) blinded outcome assessment, 32 (21.5%) demonstrated low reporting bias by preregistering or making their study protocol available and by reporting all the primary outcomes. Approximately one-fourth of all ratings (25%, 145 out of 580; 580 = total number of ratings conducted) were unclear or characterized as having high risk of bias.

A11

Full list of included articles

Physical health

Ali, L., Wallström, S., Fors, A., Barenfeld, E., Fredholm, E., Fu, M., Goudarzi, M., Gyllensten, H., Lindström Kjellberg, I., Swedberg, K., Vanfleteren, L. E. G. W., & Ekman, I. (2021). Effects of person-centered care using a digital platform and structured telephone support for people with chronic obstructive pulmonary disease and chronic heart failure: Randomized controlled trial. *Journal of Medical Internet Research*, *23*(12), e26794. https://doi.org/10.2196/26794

An, L. C., Demers, M. R. S., Kirch, M. A., Considine-Dunn, S., Nair, V., Dasgupta, K., Narisetty, N., Resnicow, K., & Ahluwalia, J. (2013). A randomized trial of an avatar-hosted multiple behavior change intervention for young adult smokers. *Journal of the National Cancer Institute Monographs*, *2013*(47), 209–215. <https://doi.org/10.1093/jncimonographs/lgt021>

Anderson, L. M. (2022). *Acceptability, feasibility, and preliminary efficacy of emphasizing peer relationships in a Facebook-based behavioral weight loss intervention for college students*. Bowling Green State University.

Baumel, A., Tinkelman, A., Mathur, N., & Kane, J. M. (2018). Digital peer-support platform (7Cups) as an adjunct treatment for women with postpartum depression: Feasibility, acceptability, and preliminary efficacy study. *JMIR mHealth and uHealth*, *6*(2), e9482. https://doi.org/[10.2196/mhealth.9482](https://doi.org/10.2196/mhealth.9482)

Bautista, C. L., Ralston, A. L., Brock, R. L., & Hope, D. A. (2022). Peer coach support in internet-based cognitive behavioral therapy for college students with social anxiety disorder: Efficacy and acceptability. *Cogent Psychology*, *9*(1), 2040160. <https://doi.org/10.1080/23311908.2022.2040160>

Birrell, L., Debenham, J., Furneaux-Bate, A., Prior, K., Spallek, S., Thornton, L., Chapman, C., & Newton, N. (2023). Evaluating a peer-support mobile app for mental health and substance use among adolescents over 12 months during the covid-19 pandemic: Randomized controlled trial. *Journal of Medical Internet Research*, *25*(3), e45216. <https://doi.org/10.2196/45216>

Bravata, D. M., Kim, J., Russell, D. W., Goldman, R., & Pace, E. (2023). Digitally enabled peer support intervention to address loneliness and mental health: Prospective cohort analysis. *JMIR Formative Research*, *7*, e48864. <https://doi.org/10.2196/48864>

Carlsen, E. M., Kyhnaeb, A., Renault, K. M., Cortes, D., Michaelsen, K. F., & Pryds, O. (2013). Telephone-based support prolongs breastfeeding duration in obese women: A randomized trial. *The American Journal of Clinical Nutrition*, *98*(5), 1226-1232. <https://doi.org/10.3945/ajcn.113.059600>

DeBar, L. L., Dickerson, J., Clarke, G., Stevens, V. J., Ritenbaugh, C., & Aickin, M. (2008). Using a website to build community and enhance outcomes in a group, multi-component intervention promoting healthy diet and exercise in adolescents. *Journal of Pediatric Psychology*, *34*(5), 539-550. <https://doi.org/10.1093%2Fjpepsy%2Fjsn126>

Dennis, C.-L., Hodnett, E., Kenton, L., Weston, J., Zupancic, J., Stewart, D. E., & Kiss, A. (2009). Effect of peer support on prevention of postnatal depression among high-risk women: Multisite randomised controlled trial. *BMJ*, *338*(7689), 280–284. <https://doi.org/10.1136/bmj.a3064>

Dennison, L., Morrison, L., Lloyd, S., Phillips, D., Stuart, B., Williams, S., Bradbury, K., Roderick, P., Murray, E., Michie, S., Little, P., & Yardley, L. (2014). Does brief telephone support improve engagement with a web-based weight management intervention? Randomized controlled trial. *Journal of Medical Internet Research*, *16*(3), e95. <https://doi.org/10.2196/jmir.3199>

Donovan, E., Martin, S. R., Seidman, L. C., Zeltzer, L. K., Cousineau, T. M., Payne, L. A., Trant, M., Weiman, M., Knoll, M., & Federman, N. C. (2019). A mobile-based mindfulness and social support program for adolescents and young adults with sarcoma: Development and pilot testing. *JMIR mHealth and uHealth*, *7*(3), e10921–e10921. <https://doi.org/10.2196/10921>

Drysdale, M. T., McBeath, M. L., & Callaghan, S. A. (2022). The feasibility and impact of online peer support on the well-being of higher education students. *The Journal of Mental Health Training, Education and Practice*, *17*(3), 206-217. <https://doi.org/10.1108/JMHTEP-02-2021-0012>

Ebert, D. D., Gollwitzer, M., Riper, H., Cuijpers, P., Baumeister, H., & Berking, M. (2013). For whom does it work? Moderators of outcome on the effect of a transdiagnostic internet-based maintenance treatment after inpatient psychotherapy: Randomized controlled trial. *Journal of Medical Internet Research*, *15*(10), e191. <https://doi.org/10.2196/jmir.2511>

Ebert, D., Tarnowski, T., Gollwitzer, M., Sieland, B., & Berking, M. (2013). A transdiagnostic internet-based maintenance treatment enhances the stability of outcome after inpatient cognitive behavioral therapy: A randomized controlled trial. *Psychotherapy and Psychosomatics*, *82*(4), 246–256. <https://doi.org/10.1159/000345967>

Economides, M., Ranta, K., Nazander, A., Hilgert, O., Goldin, P. R., Raevuori, A., & Forman-Hoffman, V. (2019). Long-term outcomes of a therapist-supported, smartphone-based intervention for elevated symptoms of depression and anxiety: Quasiexperimental, pre-postintervention study. *JMIR mHealth and uHealth*, *7*(8), e14284. <https://doi.org/10.2196%2F14284>

Fiks, A. G., Gruver, R. S., Bishop-Gilyard, C. T., Shults, J., Virudachalam, S., Suh, A. W., ... & Power, T. J. (2017). A social media peer group for mothers to prevent obesity from infancy: The Grow2Gether randomized trial. *Childhood Obesity*, *13*(5), 356-368. <https://doi.org/10.1089/chi.2017.0042>

Fortuna, K. L., Brooks, J. M., Myers, A., Sivakumar, B., & Lebby, S. R. (2022). Effectiveness of a digital peer support training program designed for rapid uptake among peer support specialists pilot study. *Psychiatric Quarterly*, *93*(3), 883-890. <https://doi.org/10.1007/s11126-022-09997-0>

Freeman, E., Barker, C., & Pistrang, N. (2008). Outcome of an online mutual support group for college students with psychological problems. *Cyberpsychology & Behavior*, *11*(5), 591-593. <https://doi.org/10.1089/cpb.2007.0133>

Gillard, S., Bremner, S., Patel, A., Goldsmith, L., Marks, J., Foster, R., ... & Priebe, S. (2022). Peer support for discharge from inpatient mental health care versus care as usual in England (ENRICH): A parallel, two-group, individually randomised controlled trial. *The Lancet Psychiatry*, *9*(2), 125-136. <https://doi.org/10.1016/s2215-0366(21)00398-9>

Gliddon, E., Cosgrove, V., Berk, L., Lauder, S., Mohebbi, M., Grimm, D., Dodd, S., Coulson, C., Raju, K., Suppes, T., & Berk, M. (2019). A randomized controlled trial of MoodSwings 2.0: An internet‐based self‐management program for bipolar disorder. *Bipolar Disorders*, *21*(1), 28–39. <https://doi.org/10.1111/bdi.12669>

Goldberg, L., Lockwood, C., Garg, B., & Kuehl, K. S. (2015). Healthy team healthy U: A prospective validation of an evidence-based worksite health promotion and wellness platform. *Frontiers in Public Health*, *3*, 155022. <https://doi.org/10.3389%2Ffpubh.2015.00188>

Gould, C. E., Carlson, C., Alfaro, A. J., Chick, C. F., Bruce, M. L., & Forman-Hoffman, V. L. (2021). Changes in quality of life and loneliness among middle-aged and older adults participating in therapist-guided digital mental health intervention. *Frontiers in Public Health*, *9*, 746904. <https://doi.org/10.3389/fpubh.2021.746904>

Grégoire, S., Beaulieu, F., Lachance, L., Bouffard, T., Vezeau, C., & Perreault, M. (2022). An online peer support program to improve mental health among university students: A randomized controlled trial. *Journal of American College Health*, *72*(7), 2001-2013. <https://doi.org/10.1080/07448481.2022.2099224>

Gulec, H., Moessner, M., Túry, F., Fiedler, P., Mezei, A., & Bauer, S. (2014). A randomized controlled trial of an internet-based posttreatment care for patients with eating disorders. *Telemedicine and e-Health*, *20*(10), 916-922. <https://doi.org/10.1089/tmj.2013.0353>

Hageman, P. A., Pullen, C. H., Hertzog, M., Pozehl, B., Eisenhauer, C., & Boeckner, L. S. (2017). Web-based interventions alone or supplemented with peer-led support or professional email counseling for weight loss and weight maintenance in women from rural communities: Results of a clinical trial. *Journal of Obesity*, *2017*, 1602627–1602621. <https://doi.org/10.1155/2017/1602627>

Harvey‐Berino, J., Pintauro, S., Buzzell, P., & Gold, E. C. (2004). Effect of internet support on the long‐term maintenance of weight loss. *Obesity Research*, *12*(2), 320-329. <https://doi.org/10.1038/oby.2004.40>

Hensel, J. M., Shaw, J., Ivers, N. M., Desveaux, L., Vigod, S. N., Bouck, Z., Onabajo, N., Agarwal, P., Mukerji, G., Yang, R., Nguyen, M., Jeffs, L., Jamieson, T., & Bhatia, R. S. (2019). Extending access to a web-based mental health intervention: Who wants more, what happens to use over time, and is it helpful? Results of a concealed, randomized controlled extension study. *BMC Psychiatry*, *19*(1), 1-10. <https://doi.org/10.1186/s12888-019-2030-x>

Hensel, J. M., Shaw, J., Ivers, N. M., Desveaux, L., Vigod, S. N., Cohen, A., ... & Bhatia, R. S. (2019). A web-based mental health platform for individuals seeking specialized mental health care services: Multicenter pragmatic randomized controlled trial. *Journal of Medical Internet Research*, *21*(6), e10838. <https://doi.org/10.2196/10838>

Houston, T. K., Cooper, L. A., & Ford, D. E. (2002). Internet support groups for depression: A 1-year prospective cohort study. *American Journal of Psychiatry*, *159*(12), 2062-2068. <https://doi.org/10.1176/appi.ajp.159.12.2062>

Imanaka, M., Ando, M., Kitamura, T., & Kawamura, T. (2013). Effectiveness of web-based self-disclosure peer-to-peer support for weight loss: Randomized controlled trial. *Journal of Medical Internet Research*, *15*(7), e136. <https://doi.org/10.2196/jmir.2405>

Johnson, F., & Wardle, J. (2011). The association between weight loss and engagement with a web-based food and exercise diary in a commercial weight loss programme: A retrospective analysis. *International Journal of Behavioral Nutrition and Physical Activity*, *8*, 1-7. <https://doi.org/10.1186/1479-5868-8-83>

Joyce, S., Shand, F., Bryant, R. A., Lal, T. J., & Harvey, S. B. (2018). Mindfulness-based resilience training in the workplace: Pilot study of the internet-based Resilience@ Work (RAW) mindfulness program. *Journal of Medical Internet Research*, *20*(9), e10326. https://doi.org/10.2196/10326

Kahl, B. L., Miller, H. M., Cairns, K., Giniunas, H., & Nicholas, M. (2020). Evaluation of ReachOut.com, an unstructured digital youth mental health intervention: Prospective cohort study. *JMIR Mental Health*, *7*(10), e21280. <https://doi.org/10.2196/21280>

Kaplan, K., Salzer, M. S., Solomon, P., Brusilovskiy, E., & Cousounis, P. (2011). Internet peer support for individuals with psychiatric disabilities: A randomized controlled trial. *Social Science & Medicine*, *72*(1), 54-62. <https://doi.org/10.1016/j.socscimed.2010.09.037>

Kelly, P. J., Beck, A. K., Deane, F. P., Larance, B., Baker, A. L., Hides, L., Manning, V., Shakeshaft, A., Neale, J., Kelly, J. F., Oldmeadow, C., Searles, A., Palazzi, K., Lawson, K., Treloar, C., Gray, R. M., Argent, A., & McGlaughlin, R. (2021). Feasibility of a mobile health app for routine outcome monitoring and feedback in SMART recovery mutual support groups: Stage 1 mixed methods pilot study. *Journal of Medical Internet Research*, *23*(10), e25217. <https://doi.org/10.2196/25217>

Keyserling, T. C., Hodge, C. D. S., Jilcott, S. B., Johnston, L. F., Garcia, B. A., Gizlice, Z., ... & Ammerman, A. S. (2008). Randomized trial of a clinic-based, community-supported, lifestyle intervention to improve physical activity and diet: The North Carolina enhanced WISEWOMAN project. *Preventive Medicine*, *46*(6), 499-510. <https://doi.org/10.1016/j.ypmed.2008.02.011>

Klimczak, K. S., Twohig, M. P., Peacock, G. G., & Levin, M. E. (2023). Using peer-support coaching to improve adherence to online ACT self-help for college mental health: A randomized controlled trial. *Behaviour Research and Therapy*, *160*, e104228. https://doi.org/10.1016/j.brat.2022.104228

Kramer, J., Boon, B., Schotanus-Dijkstra, M., van Ballegooijen, W., Kerkhof, A., & van der Poel, A. (2015). The mental health of visitors of web-based support forums for bereaved by suicide. *Crisis: The Journal of Crisis Intervention and Suicide Prevention*, *36*(1), 38–45. <https://doi.org/10.1027/0227-5910/a000281>

Krendl, A. C., & Pescosolido, B. A. (2020). Countries and cultural differences in the stigma of mental illness: The East–West divide. *Journal of Cross-Cultural Psychology, 51*(2), 149–167. https://doi.org/10.1177/0022022119901297

Kruzan, K. P., Whitlock, J., Bazarova, N. N., Bhandari, A., & Chapman, J. (2022). Use of a mobile peer support app among young people with nonsuicidal self-injury: Small-scale randomized controlled trial. *JMIR Formative Research*, *6*(1), e26526. <https://doi.org/10.2196/26526>

Lara, J., O'Brien, N., Godfrey, A., Heaven, B., Evans, E. H., Lloyd, S., Moffatt, S., Moynihan, P. J., Meyer, T. D., Rochester, L., Sniehotta, F. F., White, M., & Mathers, J. C. (2016). Pilot randomised controlled trial of a web-based intervention to promote healthy eating, physical activity and meaningful social connections compared with usual care control in people of retirement age recruited from workplaces. *PloS One*, *11*(7), e0159703. <https://doi.org/10.1371/journal.pone.0159703>

Lauder, S., Chester, A., Castle, D., Dodd, S., Gliddon, E., Berk, L., Chamberlain, J., Klein, B., Gilbert, M., Austin, D. W., & Berk, M. (2015). A randomized head to head trial of MoodSwings.net.au: An internet based self-help program for bipolar disorder. *Journal of Affective Disorders*, *171,* 13-21. <https://doi.org/10.1016/j.jad.2014.08.008>

Leahey, T. M., Fava, J. L., Seiden, A., Fernandes, D., Doyle, C., Kent, K., La Rue, M., Mitchell, M., & Wing, R. R. (2016). A randomized controlled trial testing an internet delivered cost–benefit approach to weight loss maintenance. *Preventive Medicine*, *92*, 51-57. <https://doi.org/10.1016/j.ypmed.2016.04.013>

Lee, M. K., & Suh, S.-R. (2018). Effects of peer-led interventions for patients with cancer: A meta-analysis. *Oncology Nursing Forum*, *45*(2), 217–236. <https://doi.org/10.1188/18.onf.217-236>

Lepore, S. J., Rincon, M. A., Buzaglo, J. S., Golant, M., Lieberman, M. A., Bauerle Bass, S., & Chambers, S. (2019). Digital literacy linked to engagement and psychological benefits among breast cancer survivors in Internet‐based peer support groups. *European Journal of Cancer Care*, *28*(4), e13134. https://doi.org/10.1111/ecc.13134.

Li, W., Shen, S., Yang, J., & Tang, Q. (2021). Internet-based medical service use and eudaimonic well-being of urban older adults: A peer support and technology acceptance model. *International Journal of Environmental Research and Public Health*, *18*(22), 12062. <https://doi.org/10.3390/ijerph182212062>

Lim, S. L., Johal, J., Ong, K. W., Han, C. Y., Chan, Y. H., Lee, Y. M., & Loo, W. M. (2020). Lifestyle intervention enabled by mobile technology on weight loss in patients with nonalcoholic fatty liver disease: Randomized controlled trial. *JMIR mHealth and uHealth*, *8*(4), e14802. <https://doi.org/10.2196/14802>

Linke, S., Murray, E., Butler, C., & Wallace, P. (2007). Internet-based interactive health intervention for the promotion of sensible drinking: Patterns of use and potential impact on members of the general public. *Journal of Medical Internet Research*, *9*(2), e573. <https://doi.org/10.2196%2Fjmir.9.2.e10>

Mamede, A., Noordzij, G., Jongerling, J., Snijders, M., Schop-Etman, A., & Denktas, S. (2021). Combining web-based gamification and physical nudges with an app (MoveMore) to promote walking breaks and reduce sedentary behavior of office workers: Field study. *Journal of Medical Internet Research*, *23*(4), e19875. <https://doi.org/10.2196/19875>

Marinova, N., Rogers, T., & MacBeth, A. (2022). Predictors of adolescent engagement and outcomes–a cross-sectional study using the togetherall (formerly big white wall) digital mental health platform. *Journal of Affective Disorders*, *311*, 284-293. https://doi.org/10.1016/j.jad.2022.05.058

McKay, H. G., King, D., Eakin, E. G., Seeley, J. R., & Glasgow, R. E. (2001). The diabetes network internet-based physical activity intervention: A randomized pilot study. *Diabetes Care*, *24*(8), 1328-1334. https://doi.org/10.2337/diacare.24.8.1328

McKeon, G., Steel, Z., Wells, R., Newby, J., Hadzi-Pavlovic, D., Vancampfort, D., & Rosenbaum, S. (2021). A mental health–informed physical activity intervention for first responders and their partners delivered using Facebook: Mixed methods pilot study. *JMIR Formative Research*, *5*(4), e23432. https://doi.org/10.2196/23432

Mi, R. Z., Kornfield, R., Shah, D. V., Maus, A., & Gustafson, D. H. (2022). Intraindividual, dyadic, and network communication in a digital health intervention: Distinguishing message exposure from message production. *Health Communication*, *37*(4), 397–408. <https://doi.org/10.1080/10410236.2020.1846273>

Moir, F., Henning, M., Hassed, C., Moyes, S. A., & Elley, C. R. (2016). A peer-support and mindfulness program to improve the mental health of medical students. *Teaching and Learning in Medicine*, *28*(3), 293–302. <https://doi.org/10.1080/10401334.2016.1153475>

Moravcová, K., Karbanová, M., Bretschneider, M. P., Sovová, M., Ožana, J., & Sovová, E. (2022). Comparing digital therapeutic intervention with an intensive obesity management program: Randomized controlled trial. *Nutrients*, *14*(10), 2005. <https://doi.org/10.3390/nu14102005>

Morgan, P. J., Lubans, D. R., Collins, C. E., Warren, J. M., & Callister, R. (2011). 12-month outcomes and process evaluation of the SHED-IT RCT: An internet-based weight loss program targeting men. *Obesity*, *19*(1), 142–151. <https://doi.org/10.1038/oby.2010.119>

Morriss, R., Kaylor-Hughes, C., Rawsthorne, M., Coulson, N., Simpson, S., Guo, B., James, M., Lathe, J., Moran, P., Tata, L. J., & Williams, L. (2021). A direct-to-public peer support program (big white wall) versus web-based information to aid the self-management of depression and anxiety: Results and challenges of an automated randomized controlled trial. *Journal of Medical Internet Research*, *23*(4), e23487. <https://doi.org/10.2196/23487>

Mouthaan, J., Sijbrandij, M., de Vries, G.-J., Reitsma, J. B., van de Schoot, R., Goslings, J. C., Luitse, J. S. K., Bakker, F. C., Gersons, B. P. R., & Olff, M. (2013). Internet-based early intervention to prevent posttraumatic stress disorder in injury patients: Randomized controlled trial. *Journal of Medical Internet Research*, *15*(8), e165. <https://doi.org/10.2196/jmir.2460>

Nosek, M. A., Robinson-Whelen, S., Hughes, R. B., & Nosek, T. M. (2016). An Internet-based virtual reality intervention for enhancing self-esteem in women with disabilities: Results of a feasibility study. *Rehabilitation Psychology*, *61*(4), 358–370. <https://doi.org/10.1037/rep0000107>

Owen, J. E., Curran, M., Bantum, E. O. C., & Hanneman, R. (2016). Characterizing social networks and communication channels in a web-based peer support intervention. *Cyberpsychology, Behavior, and Social Networking*, *19*(6), 388-396. <https://doi.org/10.1089/cyber.2015.0359>

Pappa, G. L., Cunha, T. O., Bicalho, P. V., Ribeiro, A., Couto Silva, A. P., Meira, W., Jr, & Beleigoli, A. M. (2017). Factors associated with weight change in online weight management communities: A case study in the lose it reddit community. *Journal of Medical Internet Research*, *19*(1), e17. <https://doi.org/10.2196/jmir.5816>

Pauksztat, B., Grech, M. R., & Kitada, M. (2022). The impact of the COVID-19 pandemic on seafarers’ mental health and chronic fatigue: Beneficial effects of onboard peer support, external support and Internet access. *Marine Policy*, *137*, e104942. <https://doi.org/10.1016/j.marpol.2021.104942>

Paulson, L. R., Casile, W. J., & Jones, D. (2015). Tech it out: Implementing an online peer consultation network for rural mental health professionals. *Journal of Rural Mental Health*, *39*(3-4), 125–136. https://doi.org/10.1037/rmh0000034

Pavarini, G., Reardon, T., Hollowell, A., Bennett, V., Lawrance, E., Pinfold, V., Singh, I., & Group, P. S. Y. P. A. (2023). Online peer support training to promote adolescents’ emotional support skills, mental health and agency during COVID-19: Randomised controlled trial and qualitative evaluation. *European Child & Adolescent Psychiatry*, *32*(6), 1119–1130. https://doi.org/10.1007//s00787-021-01933-0

Pfeiffer, P. N., Pope, B., Houck, M., Benn-Burton, W., Zivin, K., Ganoczy, D., Kim, H. M., Walters, H., Emerson, L., Nelson, C. B., Abraham, K. M., & Valenstein, M. (2020). Effectiveness of peer-supported computer-based CBT for depression among veterans in primary care. *Psychiatric Services*, *71*(3), 256–262. https://doi.org/10.1176/appi.ps.201900283

Pretlow, R. A., Stock, C. M., Allison, S., & Roeger, L. (2015). Treatment of child/adolescent obesity using the addiction model: A smartphone app pilot study. *Childhood Obesity*, *11*(3), 248–259. https://doi.org/10.1089/chi.2014.0124

Pretorius, N., Arcelus, J., Beecham, J., Dawson, H., Doherty, F., Eisler, I., ... & Schmidt, U. (2009). Cognitive-behavioural therapy for adolescents with bulimic symptomatology: The acceptability and effectiveness of internet-based delivery. *Behaviour Research and Therapy*, *47*(9), 729-736. https://doi.org/10.1016/j.brat.2009.05.006

Proudfoot, J., Parker, G., Manicavasagar, V., Hadzi-Pavlovic, D., Whitton, A., Nicholas, J., Smith, M., & Burckhardt, R. (2012). Effects of adjunctive peer support on perceptions of illness control and understanding in an online psychoeducation program for bipolar disorder: A randomised controlled trial. *Journal of Affective Disorders*, *142*(1-3), 98–105. <https://doi.org/10.1016/j.jad.2012.04.007>

Richardson, C. R., Buis, L. R., Janney, A. W., Goodrich, D. E., Sen, A., Hess, M. L., Mehari, K. S., Fortlage, L. A., Resnick, P. J., Zikmund-Fisher, B. J., Strecher, V. J., & Piette, J. D. (2010). An online community improves adherence in an internet-mediated walking program. Part 1: Results of a randomized controlled trial. *Journal of Medical Internet Research*, *12*(4), e71. <https://doi.org/10.2196/jmir.1338>

Rosas, L. G., Lv, N., Xiao, L., Azar, K. M., Hooker, S. P., Venditti, E. M., Lewis, M. A., Zavella, P., & Ma, J. (2022). Preferences for technology-mediated behavioral lifestyle interventions with different levels of coach and peer support among Latino men: Comparative study within one arm of a randomized controlled trial. *JMIR Formative Research*, *6*(2), e29537. <https://doi.org/10.2196/29537>

Ross, J. A. D., Barron, E., McGough, B., Valabhji, J., Daff, K., Irwin, J., ... & Murray, E. (2022). Uptake and impact of the English National Health Service digital diabetes prevention programme: Observational study. *BMJ Open Diabetes Research and Care*, *10*(3), e002736. <https://doi.org/10.1136/bmjdrc-2021-002736>

Salzer, M. S., Palmer, S. C., Kaplan, K., Brusilovskiy, E., Ten Have, T., Hampshire, M., Metz, J., & Coyne, J. C. (2010). A randomized, controlled study of Internet peer-to-peer interactions among women newly diagnosed with breast cancer. *Psycho-oncology*, *19*(4), 441–446. <https://doi.org/10.1002/pon.1586>

Schepens Niemiec, S. L., Blanchard, J., Vigen, C. L. P., Martínez, J., Guzmán, L., Concha, A., Fluke, M., & Carlson, M. (2018). Evaluation of ¡Vivir Mi Vida! to improve health and wellness of rural-dwelling, late middle-aged Latino adults: Results of a feasibility and pilot study of a lifestyle intervention. *Primary Health Care Research & Development*, *19*(5), 448–463. <https://doi.org/10.1017/S1463423617000901>

Schulz, A., Stolz, T., Vincent, A., Krieger, T., Andersson, G., & Berger, T. (2016). A sorrow shared is a sorrow halved? A three-arm randomized controlled trial comparing internet-based clinician-guided individual versus group treatment for social anxiety disorder. *Behaviour Research and Therapy*, *84*, 14-26. <https://doi.org/10.1016/j.brat.2016.07.001>

Sepah, S. C., Jiang, L., Ellis, R. J., McDermott, K., & Peters, A. L. (2017). Engagement and outcomes in a digital diabetes prevention program: 3-year update. *BMJ Open Diabetes Research & Care*, *5*(1), e000422. <https://doi.org/10.1136/bmjdrc-2017-000422>

Setoyama, Y., Yamazaki, Y., & Namayama, K. (2011). Benefits of peer support in online Japanese breast cancer communities: Differences between lurkers and posters. *Journal of Medical Internet Research*, *13*(4), e122. https://doi.org/10.2196/jmir.1696

Sharps, M. A., Hetherington, M. M., Blundell-Birtill, P., Rolls, B. J., & Evans, C. E. (2019). The effectiveness of a social media intervention for reducing portion sizes in young adults and adolescents. *Digital Health*, *5*, 2055207619878076. <https://doi.org/10.1177/2055207619878076>

Shorey, S., Chee, C. Y. I., Ng, E. D., Lau, Y., Dennis, C. L., & Chan, Y. H. (2019). Evaluation of a technology-based peer-support intervention program for preventing postnatal depression (Part 1): Randomized controlled trial. *Journal of Medical Internet Research*, *21*(8), e12410. <https://doi.org/10.2196/12410>

Sin, J., Henderson, C., Elkes, J., Cornelius, V., Woodham, L. A., Batchelor, R., Chen, T., Corredor, A. M., Coughlan, D., Dhital, R., Evans, S., Haider, B., Heathcote, J., Mansfield, S., O'Brien, A., Qassim, M., Sserunkuma, J., Travis, C. H., Williams, E., & Gillard, S. (2022). Effect of digital psychoeducation and peer support on the mental health of family carers supporting individuals with psychosis in England (COPe-support): A randomised clinical trial. *The Lancet Digital Health*, *4*(5), e320–e329. <https://doi.org/10.1016/s2589-7500(22)00031-0>

Smit, D., Vrijsen, J. N., Broekman, T., Groeneweg, B., & Spijker, J. (2022). User engagement within an online peer support community (depression connect) and recovery-related changes in empowerment: Longitudinal user survey. *JMIR Formative Research*, *6*(11), e39912. <https://doi.org/10.2196/39912>

Smith, D. J., Griffiths, E., Poole, R., di Florio, A., Barnes, E., Kelly, M. J., Craddock, N., Hood, K., & Simpson, S. (2011). Beating bipolar: Exploratory trial of a novel Internet-based psychoeducational treatment for bipolar disorder. *Bipolar Disorders*, *13*(5-6), 571–577. <https://doi.org/10.1111/j.1399-5618.2011.00949.x>

Stevens, M., Cartagena Farías, J., Mindel, C., D’Amico, F., & Evans-Lacko, S. (2022). Pilot evaluation to assess the effectiveness of youth peer community support via the Kooth online mental wellbeing website. *BMC Public Health*, *22*(1), 1903. <https://doi.org/https:/10.1186/s12889-022-14223-4>

Suffoletto, B., Goldstein, T., Gotkiewicz, D., Gotkiewicz, E., George, B., & Brent, D. (2021). Acceptability, engagement, and effects of a mobile digital intervention to support mental health for young adults transitioning to college: Pilot randomized controlled trial. *JMIR Formative Research*, *5*(10), e32271. <https://doi.org/10.2196/32271>

Summers, C., Tobin, S., & Unwin, D. (2021). Evaluation of the low carb program digital intervention for the self-management of Type 2 diabetes and prediabetes in an NHS England general practice: Single-arm prospective study. *JMIR Diabetes*, *6*(3), e25751. <https://doi.org/10.2196/25751>

Tate, D. F., Jackvony, E. H., & Wing, R. R. (2006). A randomized trial comparing human e-mail counseling, computer-automated tailored counseling, and no counseling in an Internet weight loss program. *Archives of Internal Medicine*, *166*(15), 1620–1625. <https://doi.org/10.1001/archinte.166.15.1620>

Tate, D. F., Wing, R. R., & Winett, R. A. (2001). Using Internet technology to deliver a behavioral weight loss program. J*AMA*, *285*(9), 1172–1177. <https://doi.org/10.1001/jama.285.9.1172>

Tomasino, K. N., Lattie, E. G., Ho, J., Palac, H. L., Kaiser, S. M., & Mohr, D. C. (2017). Harnessing peer support in an online intervention for older adults with depression. *The American Journal of Geriatric Psychiatry*, *25*(10), 1109-1119. https://doi.org/10.1016/j.jagp.2017.04.015

Travis, J., Roeder, K., Walters, H., Piette, J., Heisler, M., Ganoczy, D., Valenstein, M., & Pfeiffer, P. (2010). Telephone-based mutual peer support for depression: A pilot study. *Chronic Illness*, *6*(3), 183–191. https://doi.org/10.1177/1742395310369570

Tsai, Y. C., & Liu, C. H. (2015). An eHealth education intervention to promote healthy lifestyles among nurses. *Nursing Outlook*, *63*(3), 245–254. <https://doi.org/10.1016/j.outlook.2014.11.005>

Turner-McGrievy, G. M., & Tate, D. F. (2013). Weight loss social support in 140 characters or less: Use of an online social network in a remotely delivered weight loss intervention. *Translational Behavioral Medicine*, *3*(3), 287–294. <https://doi.org/10.1007/s13142-012-0183-y>

Van der Houwen, K., Stroebe, M., Schut, H., Stroebe, W., & Van den Bout, J. (2010). Online mutual support in bereavement: An empirical examination. *Computers in Human Behavior*, *26*(6), 1519-1525. <https://doi.org/10.1016/j.chb.2010.05.019>

Watanabe-Ito, M., Kishi, E., & Shimizu, Y. (2020). Promoting healthy eating habits for college students through creating dietary diaries via a smartphone app and social media interaction: Online survey study. *JMIR mHealth and uHealth*, *8*(3), e17613. <https://doi.org/10.2196/17613>

Webber, K. H., Tate, D. F., & Michael Bowling, J. (2008). A randomized comparison of two motivationally enhanced Internet behavioral weight loss programs. *Behaviour Research and Therapy*, *46*(9), 1090–1095. https://doi.org/10.1016/j.brat.2008.06.008

West, D. S., Monroe, C. M., Turner-McGrievy, G., Sundstrom, B., Larsen, C., Magradey, K., Wilcox, S., & Brandt, H. M. (2016). A technology-mediated behavioral weight gain prevention intervention for college students: Controlled, quasi-experimental study. *Journal of Medical Internet Research*, *18*(6), e133. <https://doi.org/10.2196/jmir.5474>

Wilkerson, D. A., Brady, E., Yi, E. H., & Bateman, D. R. (2018). Friendsourcing peer support for Alzheimer’s caregivers using Facebook social media. *Journal of Technology in Human Services*, *36*(2-3), 105-124. <https://doi.org/10.1080/15228835.2018.1449709>

Wright, H., Turner, A., Ennis, S., Percy, C., Loftus, G., Clyne, W., Matouskova, G., & Martin, F. (2022). Digital peer-supported self-management intervention codesigned by people with long covid: Mixed methods proof-of-concept study. *JMIR Formative Research*, *6*(10), e41410. <https://doi.org/10.2196/41410>

Yeung, A., Xie, Q., Huang, X., Hoeppner, B., Jain, F. A., Tan, E. K., ... & Guo, X. (2021). Effectiveness of mindful self-compassion training supported by online peer groups in China: A pilot study. *Alternative Therapies in Health and Medicine*, *27*(5), 170. <https://doi.org/34559687>

Yeung, R. O., Cai, J. H., Zhang, Y., Luk, A. O., Pan, J. H., Yin, J., Ozaki, R., Kong, A. P. S., Ma, R., So, W. Y., Tsang, C. C., Lau, K. P., Fisher, E., Goggins, W., Oldenburg, B., & Chan, J. (2018). Determinants of hospitalization in Chinese patients with type 2 diabetes receiving a peer support intervention and JADE integrated care: The PEARL randomised controlled trial. *Clinical Diabetes and Endocrinology*, *4*, 1-11. <https://doi.org/10.1186/s40842-018-0055-6>

Zheng, Y., Wang, W., Zhong, Y., Wu, F., Zhu, Z., Tham, Y. C., Lamoureux, E., Xiao, L., Zhu, E., Liu, H., Jin, L., Liang, L., Luo, L., He, M., Morgan, I., Congdon, N., & Liu, Y. (2021). A peer-to-peer live-streaming intervention for children during COVID-19 homeschooling to promote physical activity and reduce anxiety and eye strain: Cluster randomized controlled trial. *Journal of Medical Internet Research*, *23*(4), e24316. <https://doi.org/10.2196/24316>

Mental health

Andersson, G., Bergström, J., Holländare, F., Carlbring, P. E. R., Kaldo, V., & Ekselius, L. (2005). Internet-based self-help for depression: Randomised controlled trial. *The British Journal of Psychiatry*, *187*(5), 456-461. https://doi.org/[10.1192/bjp.187.5.456](https://doi.org/10.1192/bjp.187.5.456)

Baumel, A., Tinkelman, A., Mathur, N., & Kane, J. M. (2018). Digital peer-support platform (7Cups) as an adjunct treatment for women with postpartum depression: Feasibility, acceptability, and preliminary efficacy study. *JMIR mHealth and uHealth*, *6*(2), e9482. https://doi.org/[10.2196/mhealth.9482](https://doi.org/10.2196/mhealth.9482)

Bautista, C. L., Ralston, A. L., Brock, R. L., & Hope, D. A. (2022). Peer coach support in internet-based cognitive behavioral therapy for college students with social anxiety disorder: Efficacy and acceptability. *Cogent Psychology*, *9*(1), 2040160. <https://doi.org/10.1080/23311908.2022.2040160>

Bilich, L. L., Deane, F. P., Phipps, A. B., Barisic, M., & Gould, G. (2008). Effectiveness of bibliotherapy self‐help for depression with varying levels of telephone helpline support. *Clinical Psychology & Psychotherapy: An International Journal of Theory & Practice*, *15*(2), 61-74. https://doi.org/[10.1002/cpp.562](https://doi.org/10.1002/cpp.562)

Birrell, L., Debenham, J., Furneaux-Bate, A., Prior, K., Spallek, S., Thornton, L., Chapman, C., & Newton, N. (2023). Evaluating a peer-support mobile app for mental health and substance use among adolescents over 12 months during the covid-19 pandemic: Randomized controlled trial. *Journal of Medical Internet Research*, *25*(3), e45216. https://doi.org/10.2196/45216

Buglione, S. A., Devito, A. J., & Mulloy, J. M. (1990). Traditional group therapy and computer-administered treatment for test anxiety. *Anxiety Research*, *3*(1), 33-39. https://doi.org/10.1080/08917779008248739

Buntrock, C., Ebert, D. D., Lehr, D., Smit, F., Riper, H., Berking, M., & Cuijpers, P. (2016). Effect of a web-based guided self-help intervention for prevention of major depression in adults with subthreshold depression: A randomized clinical trial. *JAMA*, *315*(17), 1854–1863. https://doi.org/10.1001/jama.2016.4326

Dennis, C. L. (2003). The effect of peer support on postpartum depression: A pilot randomized controlled trial. *The Canadian Journal of Psychiatry*, *48*(2), 115-124. https://doi.org/10.1177/070674370304800209

Donovan, E., Martin, S. R., Seidman, L. C., Zeltzer, L. K., Cousineau, T. M., Payne, L. A., Trant, M., Weiman, M., Knoll, M., & Federman, N. C. (2019). A mobile-based mindfulness and social support program for adolescents and young adults with sarcoma: Development and pilot testing. *JMIR mHealth and uHealth*, *7*(3), e10921–e10921. https://doi.org/10.2196/10921

Drysdale, M. T., McBeath, M. L., & Callaghan, S. A. (2022). The feasibility and impact of online peer support on the well-being of higher education students. *The Journal of Mental Health Training, Education and Practice*, *17*(3), 206-217. https://doi.org/10.1108/JMHTEP-02-2021-0012

Ebert, D. D., Gollwitzer, M., Riper, H., Cuijpers, P., Baumeister, H., & Berking, M. (2013). For whom does it work? Moderators of outcome on the effect of a transdiagnostic internet-based maintenance treatment after inpatient psychotherapy: Randomized controlled trial. *Journal of Medical Internet Research*, *15*(10), e191. https://doi.org/10.2196/jmir.2511

Ehlers, A., Clark, D. M., Hackmann, A., McManus, F., Fennell, M., Herbert, C., & Mayou, R. (2003). A randomized controlled trial of cognitive therapy, a self-help booklet, and repeated assessments as early interventions for posttraumatic stress disorder. *Archives of General Psychiatry*, *60*(10), 1024-1032. https://doi.org/10.1001/archpsyc.60.10.1024

Fortuna, K. L., Wright, A. C., Mois, G., Myers, A. L., Kadakia, A., & Collins-Pisano, C. (2022). Feasibility, acceptability, and potential utility of peer-supported ecological momentary assessment among people with serious mental illness: A pilot study. *Psychiatric Quarterly*, *93*(3), 717-735. https://doi.org/10.1007/s11126-022-09986-3

Freeman, E., Barker, C., & Pistrang, N. (2008). Outcome of an online mutual support group for college students with psychological problems. *Cyberpsychology & Behavior*, *11*(5), 591-593. https://doi.org/10.1089/cpb.2007.0133

Geraedts, A. S., Kleiboer, A. M., Twisk, J., Wiezer, N. M., van Mechelen, W., & Cuijpers, P. (2014). Long-term results of a web-based guided self-help intervention for employees with depressive symptoms: Randomized controlled trial. *Journal of Medical Internet Research*, *16*(7), e168. https://doi.org/10.2196/jmir.3539

Grégoire, S., Beaulieu, F., Lachance, L., Bouffard, T., Vezeau, C., & Perreault, M. (2022). An online peer support program to improve mental health among university students: A randomized controlled trial. *Journal of American College Health*, *72*(7), 2001-2013. https://doi.org/10.1080/07448481.2022.2099224

Hensel, J. M., Shaw, J., Ivers, N. M., Desveaux, L., Vigod, S. N., Cohen, A., ... & Bhatia, R. S. (2019). A web-based mental health platform for individuals seeking specialized mental health care services: Multicenter pragmatic randomized controlled trial. *Journal of Medical Internet Research*, *21*(6), e10838. https://doi.org/10.2196/10838

Higgins, J. P., Thompson, S. G., Deeks, J. J., & Altman, D. G. (2003). Measuring inconsistency in meta-analyses. *BMJ*, *327*(7414), 557-560. https://doi.org/10.1136/bmj.327.7414.557

Horgan, A., McCarthy, G., & Sweeney, J. (2013). An evaluation of an online peer support forum for university students with depressive symptoms. *Archives of Psychiatric Nursing*, *27*(2), 84-89. https://doi.org/10.1016/j.apnu.2012.12.005

Houston, T. K., Cooper, L. A., & Ford, D. E. (2002). Internet support groups for depression: A 1-year prospective cohort study. *American Journal of Psychiatry*, *159*(12), 2062-2068. https://doi.org/10.1176/appi.ajp.159.12.2062

Joyce, S., Shand, F., Bryant, R. A., Lal, T. J., & Harvey, S. B. (2018). Mindfulness-based resilience training in the workplace: Pilot study of the internet-based Resilience@ Work (RAW) mindfulness program. *Journal of Medical Internet Research*, *20*(9), e10326. https://doi.org/10.2196/10326

Klimczak, K. S., Twohig, M. P., Peacock, G. G., & Levin, M. E. (2023). Using peer-support coaching to improve adherence to online ACT self-help for college mental health: A randomized controlled trial. *Behaviour Research and Therapy*, *160*, e104228. https://doi.org/10.1016/j.brat.2022.104228

Kramer, J., Boon, B., Schotanus-Dijkstra, M., van Ballegooijen, W., Kerkhof, A., & van der Poel, A. (2015). The mental health of visitors of web-based support forums for bereaved by suicide. *Crisis: The Journal of Crisis Intervention and Suicide Prevention*, *36*(1), 38–45. https://doi.org/10.1027/0227-5910/a000281

Leow, M., Chan, S., & Chan, M. (2015). A pilot randomized, controlled trial of the effectiveness of a psychoeducational intervention on family caregivers of patients with advanced cancer. *Oncology Nursing Forum*, *42*(2), E63–E72. https://doi.org/10.1188/15.onf.e63-e72

Lepore, S. J., Rincon, M. A., Buzaglo, J. S., Golant, M., Lieberman, M. A., Bauerle Bass, S., & Chambers, S. (2019). Digital literacy linked to engagement and psychological benefits among breast cancer survivors in Internet‐based peer support groups. *European Journal of Cancer Care*, *28*(4), e13134. https://doi.org/10.1111/ecc.13134.

Lieberman, M. A., & Goldstein, B. A. (2005). Self-help on-line: An outcome evaluation of breast cancer bulletin boards. *Journal of Health Psychology*, *10*(6), 855-862. https://doi.org/10.1177/1359105305057319

Linke, S., Murray, E., Butler, C., & Wallace, P. (2007). Internet-based interactive health intervention for the promotion of sensible drinking: Patterns of use and potential impact on members of the general public. *Journal of Medical Internet Research*, *9*(2), e10. <https://doi.org/10.2196/jmir.9.2.e10>

Ludman, E. J., Simon, G. E., Grothaus, L. C., Luce, C., Markley, D. K., & Schaefer, J. (2007). A pilot study of telephone care management and structured disease self-management groups for chronic depression. *Psychiatric Services*, *58*(8), 1065-1072. https://doi.org/10.1176/ps.2007.58.8.1065

Marinova, N., Rogers, T., & MacBeth, A. (2022). Predictors of adolescent engagement and outcomes–a cross-sectional study using the togetherall (formerly big white wall) digital mental health platform. *Journal of Affective Disorders*, *311*, 284-293. https://doi.org/10.1016/j.jad.2022.05.058

McKay, H. G., Glasgow, R. E., Feil, E. G., Boles, S. M., & Barrera, M. (2002). Internet-based diabetes self-management and support: Initial outcomes from the diabetes network project. *Rehabilitation Psychology*, *47*(1), 31–48. https://doi.org/10.1037//0090-5550.47.1.31

McKay, H. G., King, D., Eakin, E. G., Seeley, J. R., & Glasgow, R. E. (2001). The diabetes network internet-based physical activity intervention: A randomized pilot study. *Diabetes Care*, *24*(8), 1328-1334. https://doi.org/10.2337/diacare.24.8.1328

Mouthaan, J., Sijbrandij, M., de Vries, G.-J., Reitsma, J. B., van de Schoot, R., Goslings, J. C., Luitse, J. S. K., Bakker, F. C., Gersons, B. P. R., & Olff, M. (2013). Internet-based early intervention to prevent posttraumatic stress disorder in injury patients: Randomized controlled trial. *Journal of Medical Internet Research*, *15*(8), e165. https://doi.org/10.2196/jmir.2460

Nelson, C. B., Abraham, K. M., Walters, H., Pfeiffer, P. N., & Valenstein, M. (2014). Integration of peer support and computer-based CBT for veterans with depression. *Computers in Human Behavior*, *31*, 57-64. https://doi.org/10.1016/j.chb.2013.10.012

Nosek, M. A., Robinson-Whelen, S., Hughes, R. B., & Nosek, T. M. (2016). An internet-based virtual reality intervention for enhancing self-esteem in women with disabilities: Results of a feasibility study. *Rehabilitation Psychology*, *61*(4), 358–370. https://doi.org/10.1037/rep0000107

O’Dea, B., Han, J., Batterham, P. J., Achilles, M. R., Calear, A. L., Werner‐Seidler, A., ... & Christensen, H. (2020). A randomised controlled trial of a relationship‐focussed mobile phone application for improving adolescents’ mental health. *Journal of Child Psychology and Psychiatry*, *61*(8), 899-913. https://doi.org/doi:10.1111/jcpp.13294

Owen, J. E., Curran, M., Bantum, E. O. C., & Hanneman, R. (2016). Characterizing social networks and communication channels in a web-based peer support intervention. *Cyberpsychology, Behavior, and Social Networking*, *19*(6), 388-396. https://doi.org/10.1089/cyber.2015.0359

Pauksztat, B., Grech, M. R., & Kitada, M. (2022). The impact of the COVID-19 pandemic on seafarers’ mental health and chronic fatigue: Beneficial effects of onboard peer support, external support and Internet access. *Marine Policy*, *137*, e104942. https://doi.org/10.1016/j.marpol.2021.104942

Paulson, L. R., Casile, W. J., & Jones, D. (2015). Tech it out: Implementing an online peer consultation network for rural mental health professionals. *Journal of Rural Mental Health*, *39*(3-4), 125–136. https://doi.org/10.1037/rmh0000034

Pavarini, G., Reardon, T., Hollowell, A., Bennett, V., Lawrance, E., Peer Support Young People’s Advisory Group, Pinfold, V., & Singh, I.. (2023). Online peer support training to promote adolescents’ emotional support skills, mental health and agency during COVID-19: Randomised controlled trial and qualitative evaluation. *European Child & Adolescent Psychiatry*, *32*(6), 1119–1130. https://doi.org/10.1007//s00787-021-01933-0

Pretorius, N., Arcelus, J., Beecham, J., Dawson, H., Doherty, F., Eisler, I., ... & Schmidt, U. (2009). Cognitive-behavioural therapy for adolescents with bulimic symptomatology: The acceptability and effectiveness of internet-based delivery. *Behaviour Research and Therapy*, *47*(9), 729-736. https://doi.org/10.1016/j.brat.2009.05.006

Schulz, A., Stolz, T., Vincent, A., Krieger, T., Andersson, G., & Berger, T. (2016). A sorrow shared is a sorrow halved? A three-arm randomized controlled trial comparing internet-based clinician-guided individual versus group treatment for social anxiety disorder. *Behaviour Research and Therapy*, *84*, 14-26. https://doi.org/10.1016/j.brat.2016.07.001

Setoyama, Y., Yamazaki, Y., & Namayama, K. (2011). Benefits of peer support in online Japanese breast cancer communities: Differences between lurkers and posters. *Journal of Medical Internet Research*, *13*(4), e122. https://doi.org/10.2196/jmir.1696

Sin, J., Henderson, C., Elkes, J., Cornelius, V., Woodham, L. A., Batchelor, R., Chen, T., Corredor, A. M., Coughlan, D., Dhital, R., Evans, S., Haider, B., Heathcote, J., Mansfield, S., O'Brien, A., Qassim, M., Sserunkuma, J., Travis, C. H., Williams, E., & Gillard, S. (2022). Effect of digital psychoeducation and peer support on the mental health of family carers supporting individuals with psychosis in England (COPe-support): A randomised clinical trial. *The Lancet Digital Health*, *4*(5), e320–e329. https://doi.org/10.1016/s2589-7500(22)00031-0

Stevens, M., Cartagena Farías, J., Mindel, C., D’Amico, F., & Evans-Lacko, S. (2022). Pilot evaluation to assess the effectiveness of youth peer community support via the Kooth online mental wellbeing website. *BMC Public Health*, *22*(1), 1903. https://doi.org/https:/10.1186/s12889-022-14223-4

Suffoletto, B., Goldstein, T., Gotkiewicz, D., Gotkiewicz, E., George, B., & Brent, D. (2021). Acceptability, engagement, and effects of a mobile digital intervention to support mental health for young adults transitioning to college: Pilot randomized controlled trial. *JMIR Formative Research*, *5*(10), e32271. https://doi.org/10.2196/32271

Titov, N., Andrews, G., & Schwencke, G. (2008). Shyness 2: Treating social phobia online: Replication and extension. *Australian & New Zealand Journal of Psychiatry*, *42*(7), 595-605. https://doi.org/10.1080/00048670802119820

Titov, N., Andrews, G., Schwencke, G., Drobny, J., & Einstein, D. (2008). Shyness 1: Distance treatment of social phobia over the Internet. *Australian & New Zealand Journal of Psychiatry*, *42*(7), 585-594. https://doi.org/10.1080/00048670802119762

Tomasino, K. N., Lattie, E. G., Ho, J., Palac, H. L., Kaiser, S. M., & Mohr, D. C. (2017). Harnessing peer support in an online intervention for older adults with depression. *The American Journal of Geriatric Psychiatry*, *25*(10), 1109-1119. https://doi.org/10.1016/j.jagp.2017.04.015

Travis, J., Roeder, K., Walters, H., Piette, J., Heisler, M., Ganoczy, D., Valenstein, M., & Pfeiffer, P. (2010). Telephone-based mutual peer support for depression: A pilot study. *Chronic Illness*, *6*(3), 183–191. https://doi.org/10.1177/1742395310369570

Van der Houwen, K., Stroebe, M., Schut, H., Stroebe, W., & Van den Bout, J. (2010). Online mutual support in bereavement: An empirical examination. *Computers in Human Behavior*, *26*(6), 1519-1525. https://doi.org/10.1016/j.chb.2010.05.019

Wright, H., Turner, A., Ennis, S., Percy, C., Loftus, G., Clyne, W., Matouskova, G., & Martin, F. (2022). Digital peer-supported self-management intervention codesigned by people with long covid: Mixed methods proof-of-concept study. *JMIR Formative Research*, *6*(10), e41410. https://doi.org/10.2196/41410

Yeung, A., Xie, Q., Huang, X., Hoeppner, B., Jain, F. A., Tan, E. K., ... & Guo, X. (2021). Effectiveness of mindful self-compassion training supported by online peer groups in China: A pilot study. *Alternative Therapies in Health and Medicine*, *27*(5), 170. https://doi.org/34559687

**References (cited in Online Supplement and Tables)**

Borenstein, M., Hedges, L. V., Higgins, L. V., & Rothstein, H. R. (2021). *Introduction to meta-analysis.* Wiley.

Egger, M., Smith, G. D., Schneider, M., & Minder, C. (1997). *Bias in meta-analysis detected by a simple, graphical test. BMJ, 315*(7109), 629-634. https://doi.org/10.1136/bmj.315.7109.629

Harrer, M., Cuijpers, P., Furukawa, T. A., & Ebert, D. D. (2021). *Doing meta-analysis with R: A hands-on guide.* Chapman & Hall/CRC Press.

Hunter, J. E., & Schmidt, F. L. (2004). *Methods of meta-analysis* (2nd ed.). Thousand Oaks, CA: Sage.

Hunter, J. E., & Schmidt, F. L. (2014). *Methods of meta-analysis: Correcting error and bias in research findings* (3rd ed.). Sage.

Tipton, E. (2015). Small sample adjustments for robust variance estimation with meta-regression. Psychological Methods, 20(3), 375–393. https://doi.org/10.1037/met0000011

1. Some studies examined more than one source of support and study design (i.e., pre vs. post, digital peer support vs. control, and digital peer support vs. alternative interventions) [↑](#footnote-ref-1)
2. Some studies examined more than one source of support and study design (i.e., pre vs. post, digital peer support vs. control, and digital peer support vs. alternative interventions) [↑](#footnote-ref-2)
